# Distinct colon mucosa microbiomes associated with tubular adenomas and serrated polyps

**DOI:** 10.1038/s41522-022-00328-6

**Published:** 2022-08-29

**Authors:** Julio Avelar-Barragan, Lauren DeDecker, Zachary N. Lu, Bretton Coppedge, William E. Karnes, Katrine L. Whiteson

**Affiliations:** 1 School of Biological Sciences, University of California, Irvine, CA USA; 2 School of Medicine, University of California, Irvine, CA USA

**Keywords:** Microbiome, Microbiota

## Abstract

Colorectal cancer is the second most deadly and third most common cancer in the world. Its development is heterogenous, with multiple mechanisms of carcinogenesis. Two distinct mechanisms include the adenoma-carcinoma sequence and the serrated pathway. The gut microbiome has been identified as a key player in the adenoma-carcinoma sequence, but its role in serrated carcinogenesis is less clear. In this study, we characterized the gut microbiome of 140 polyp-free and polyp-bearing individuals using colon mucosa and fecal samples to determine if microbiome composition was associated with each of the two key pathways. We discovered significant differences between the microbiomes of colon mucosa and fecal samples, with sample type explaining 10–15% of the variation observed in the microbiome. Multiple mucosal brushings were collected from each individual to investigate whether the gut microbiome differed between polyp and healthy intestinal tissue, but no differences were found. Mucosal aspirate sampling revealed that the microbiomes of individuals with tubular adenomas and serrated polyps were significantly different from each other and polyp-free individuals, explaining 1–4% of the variance in the microbiome. Microbiome composition also enabled the accurate prediction of subject polyp types using Random Forest, which produced an area under curve values of 0.87–0.99. By directly sampling the colon mucosa and distinguishing between the different developmental pathways of colorectal cancer, our study helps characterize potential mechanistic targets for serrated carcinogenesis. This research also provides insight into multiple microbiome sampling strategies by assessing each method’s practicality and effect on microbial community composition.

## Introduction

Colorectal cancer (CRC) is the second most deadly and third most common cancer globally, accounting for over 900,000 deaths in 2020^1^. The etiologies of CRC are multifactorial, with only 5–10% of cases being attributable to hereditary germline mutations^2^. Significant risk factors include diets high in red meat and low in fiber, obesity, physical inactivity, drug and alcohol usage, and chronic bowel inflammation^3–6^. Each of these factors is associated with compositional and functional changes in the collective community of bacteria, fungi, archaea, and viruses that inhabit the colon^7–10^. Commonly referred to as the gut microbiome, this community of microorganisms has been identified as a potential regulator of CRC initiation and progression.

Colorectal polyp formation precedes cancer development and is influenced by various environmental factors and host genetics. Polyps most commonly progress into malignancy through the adenoma-carcinoma sequence^11^. This pathway is characterized by chromosomal instability and mutations in the adenomatous polyposis coli (APC) gene, KRAS oncogene, and TP53 tumor suppressor gene^12^. Alternatively, 15 to 30% of CRCs develop through the serrated pathway^13^. This pathway is characterized by the epigenetic hypermethylation of gene promoters to produce a CpG island methylator phenotype^13^. In addition to the epigenetic inactivation of tumor suppressor genes, BRAF or KRAS mutations are also common^13^. The serrated pathway often results in the production of hyperplastic polyps (HPPs), traditional serrated adenomas (TSAs), and sessile serrated polyps (SSPs)^14^. Premalignant polyps from both pathways can be screened for and removed during colonoscopy to prevent CRC formation, but incomplete polyp resection or escaped detection can result in the development of interval cancers. Compared to other colorectal polyps, SSPs are disproportionately responsible for interval cancers, as their flat morphology makes them difficult to detect^15^. Additional detection methods, such as SSP-specific biomarkers, would assist with CRC prevention.

One potential avenue for polyp-specific biomarker discovery is the gut microbiota. SSPs often overexpress mucin-forming genes, like MUC6, MUC5aC, MUC17, and MUC2, producing a mucus cap, which may harbor unique, mucin-degrading microbes^16^. Finding microbiome alterations in patients consistent with the presence of SSPs would enable gastroenterologists to personalize their technique and screening frequency for these higher-risk patients. Additionally, elucidating the microbiome alterations specific to the adenoma-carcinoma sequence or the serrated pathway would help better understand the mechanisms of how particular microbes, their metabolites, and dysbiosis may contribute to colorectal carcinogenesis.

Studies comparing the microbiomes of these two pathways with healthy controls have yet to discover differences between healthy individuals and those with serrated polyps^17–19^. One reason for this may be the dominant use of stool for characterizing the microbiome, which does not accurately represent microbes adherent to the intestinal epithelium^20,21^. In this regard, we hypothesized that colon mucosa samples would more accurately reflect the composition of microbes intimately associated with colorectal polyps. To investigate this and the role of the microbiome in the adenoma-carcinoma and serrated pathways, we used multiple sampling techniques to obtain microbiome samples during and after colonoscopy from polyp-free individuals or those with tubular adenomas (TAs), HPPs, or SSPs. When possible, mucosal brush samples from the same individual were collected from polyps and the healthy colon tissue opposite from these polyps. Stool samples were also collected 4–6 weeks after colonoscopy. We used a combination of amplicon (16S and ITS) and shotgun sequencing to study the microbial communities of samples. The purpose of our work was to (1) develop and compare microbiome sampling methods during colonoscopy, (2) characterize the microbiomes of a polyp and healthy tissue samples within the same individuals, and (3) identify microbes or microbial genes specific to CRC precursors in the adenoma-carcinoma sequence versus the serrated pathway. Our key hypothesis was that there would be distinct differences between the microbiomes of individuals with tubular adenomas versus serrated polyps.

## Results

### Description of the study cohort, samples, and data collected

**Fig. 1:**
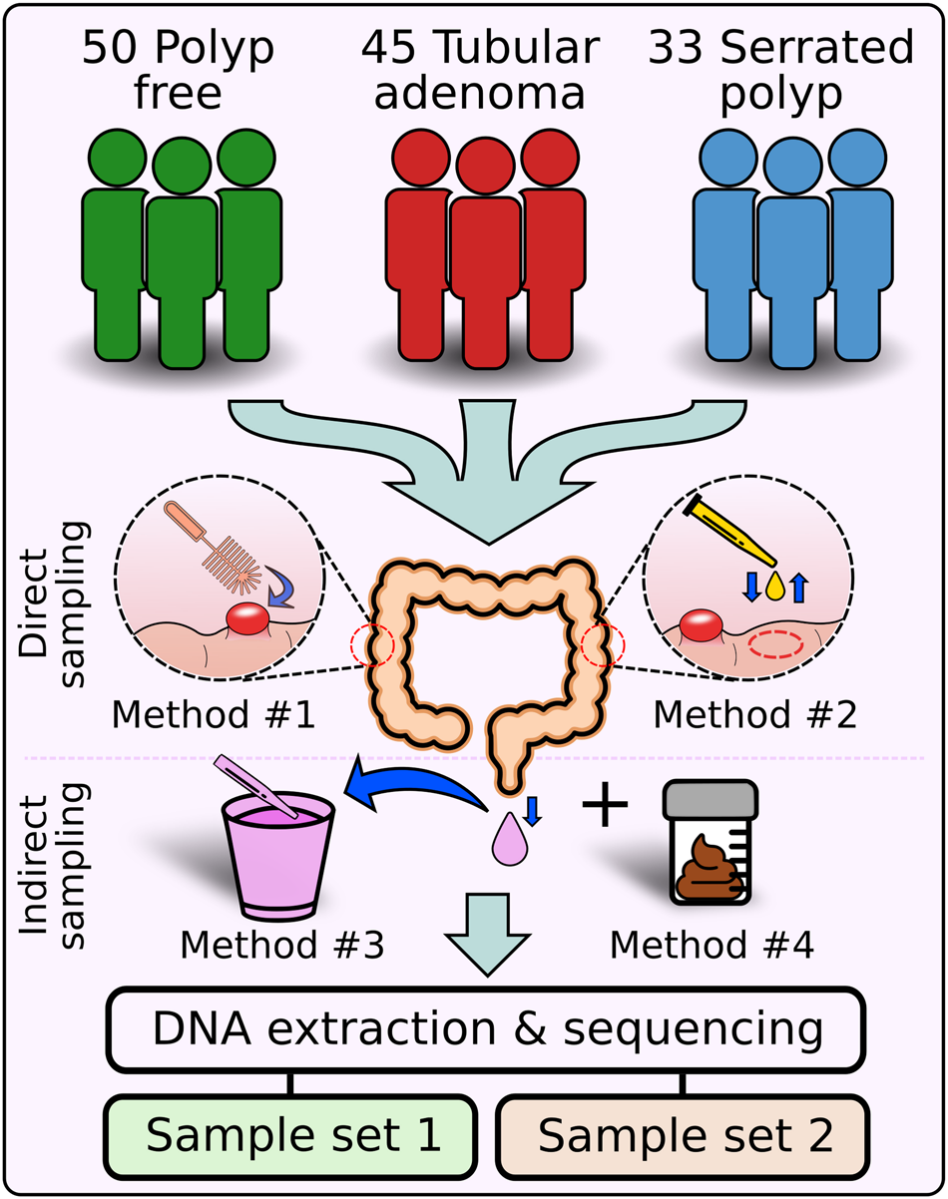
Study design. A total of 140 individuals were recruited for this study, including 50 polyp-free individuals, 45 with tubular adenomas, and 33 with serrated polyps (HPP, TSA, or SSP). The remaining 12 individuals had missing or unknown pathologies. Multiple samples were taken from each subject during colonoscopy. This included mucosal brushes (Method #1, orange), mucosal aspirates (Method #2, yellow), and lavage aspirates (Method #3, purple). Fecal samples (Method #4, brown) were collected from participants 4 to 6 weeks post-colonoscopy. DNA extraction and sequencing produced two sample sets. The first sample set was produced by sequencing mucosal brushes, mucosal aspirates, and lavage aspirates using 16S and ITS sequencing. The second sample set was produced by sequencing mucosal aspirates, lavage aspirates, and fecal samples using whole-genome shotgun sequencing.

We collected 1883 mucosal brushes, mucosal aspirates, lavage aspirates, and fecal samples from 140 individuals with and without colorectal polyps (Supplementary Table 1). Of those, 50 individuals were polyp-free, 45 had at least one tubular adenoma, and 33 had at least one serrated polyp (Fig. 1). The remaining 12 subjects had missing or unknown pathologies. We generated data from two sample sets. The first sample set was characterized using 16S and ITS sequencing, while the second sample set was analyzed using shotgun sequencing. Details on the number of samples, sample types, polyp types, and subject characteristics for each dataset can be found in Table 1 and Supplementary Tables 2–4.

**Table 1:** Study cohort information.

	Sample set 1 (16S)	Sample set 1 (ITS)	Sample set 2 (Shotgun)
Number of samples	147	98	238
Sample types	Mucosal brushes	Mucosal brushes	Mucosal aspirates
	Mucosal aspirates	Mucosal aspirates	Lavage aspirates
	Lavage aspirates	Lavage aspirates	Fecal samples
Median Age (Years)	60	61	65
Median BMI (kg/m^2^)	25	25	26
Ethnicity	White: 60%	White: 71%	White: 58%
	Black: 7%	Black: 3%	Black: 1%
	Asian: 21%	Asian: 13%	Asian: 16%
	Hispanic: 8%	Hispanic: 11%	Hispanic: 11%
	Other/Unknown: 4%	Other/Unknown: 2%	Other/Unknown: 14%
Sex	Male: 57%	Male: 63%	Male: 48%
	Female: 43%	Female: 37%	Female: 39%
	Other/Unknown: 0%	Other/Unknown: 0%	Other/Unknown: 13%

A table describing the sample sizes, sample types, median age, median BMI, ethnicity compositions, and sex ratios of each sample set. The first sample set was sequenced twice, once using 16S sequencing and once using ITS sequencing.

### Microbiomes of mucosal and lavage samples are similar to each other but different from those in feces

**Fig. 2:**
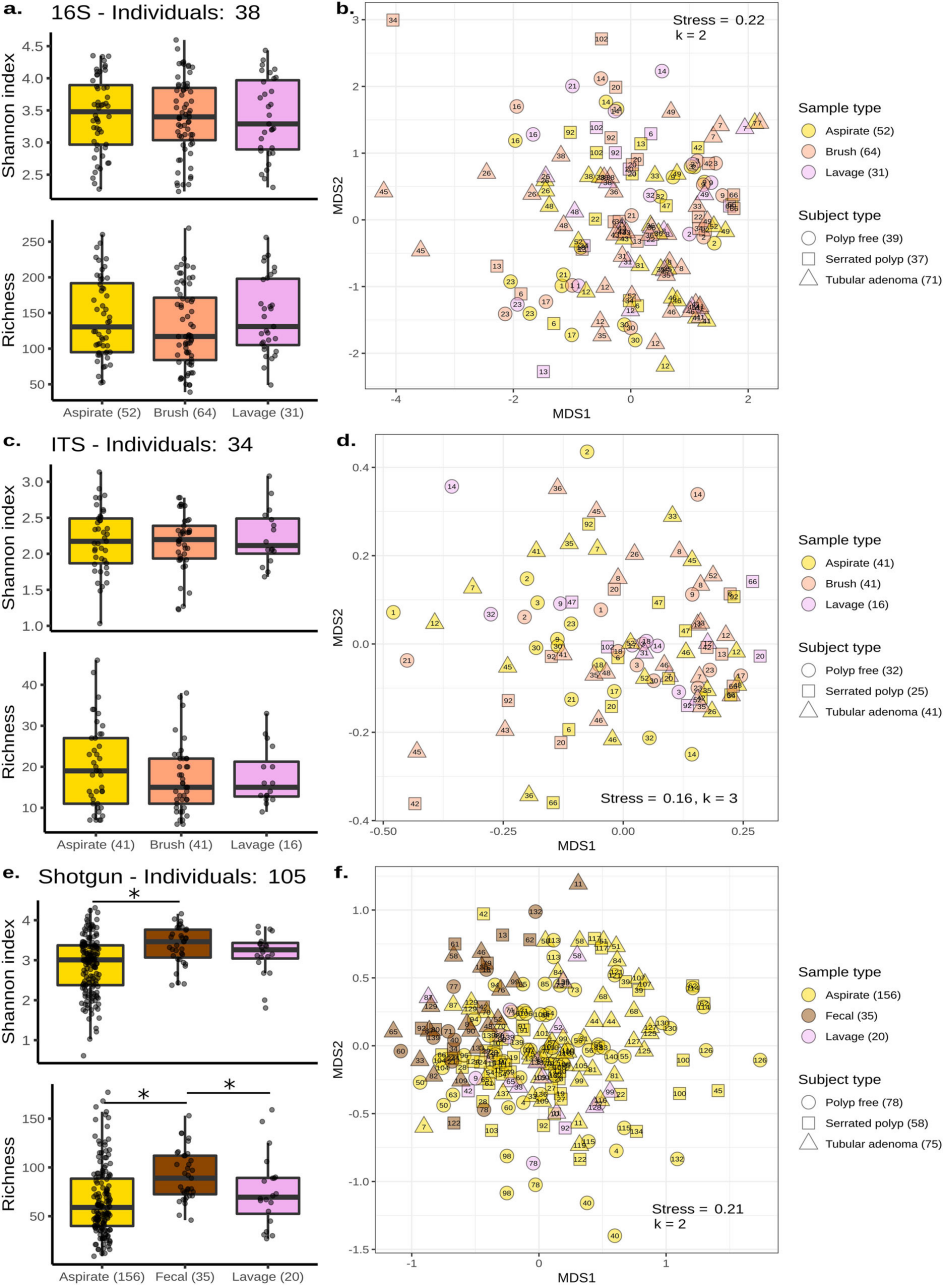
Microbiomes of mucosal and lavage samples are similar to each other but different from those in Feces. **a**, **c**, **e** Box plots showing Shannon diversity and richness estimates across mucosal aspirates (yellow), mucosal brushes (orange), lavage aspirates (purple), and fecal samples (brown). The first sample set was sequenced using 16S (**a**), and ITS (**c**) sequencing. The second sample set was sequenced using shotgun sequencing (**e**). The center line within each box defines the median, boxes define the upper and lower quartiles, and whiskers define 1.5x the interquartile range. **b**, **d**, **f** Non-metric multidimensional scaling of Bray–Curtis dissimilarities produced from 16S (**b**), ITS (**d**), and shotgun (**f**) compositional data. Each point corresponds to one sample, with multiple samples per individual. The individual of origin is denoted numerically within each point. The number of samples per sample type and subject category are annotated parenthetically. Significant comparisons (Linear-mixed effects: *p* < 0.05) are denoted with an asterisk (*). Source data are provided as a Source Data file.

Our first objective was to determine whether microbiome composition varied between sample types. We began by sequencing DNA from mucosal brushes, mucosal aspirates, and lavage aspirates from a subset of 38 individuals using 16S amplicon sequencing. Fecal samples were not included because they were collected later. We observed no significant differences in Shannon diversity or richness across mucosal brushes, mucosal aspirates, and lavage aspirates (Linear-mixed effects model, LME: *p* > 0.05, Fig. 2a). Permutational multivariate analysis of variance (PERMANOVA) on Bray–Curtis dissimilarities revealed that the individual explained the greatest amount of variation in microbiome composition (PERMANOVA: *p* = 0.001, *R*^2^ = 0.51; Supplementary Table 5). This analysis found no significant differences in the microbiomes associated with mucosal brushes, mucosal aspirates, and lavage aspirates from within the same individual (PERMANOVA: *p* = 0.99, *R*^2^ = 0.15; Supplementary Table 5). The lack of significance was consistent with no discernable clusters based on sample type (Fig. 2b). The abundances of three amplicon sequence variants (ASV) significantly differed across the three sampling methods—one from the *Gemellaceae* family and two *Streptococcus spp*. Abundances of these ASVs were higher in mucosal aspirates compared to mucosal brushes (ANCOM2: *p*-adj <0.05; Supplementary Fig. 1).

ITS2 sequencing was also performed on the same subset of samples to investigate the effect of the sampling method on the fungal microbiome. We observed no differences in Shannon diversity or richness across mucosal brushes, mucosal aspirates, and lavage aspirates (LME: *p* > 0.05, Fig. 2c). Beta-diversity ordination by sample type demonstrated no discernable clustering (Fig. 2d). Like 16S amplicon data, PERMANOVA analysis of Bray–Curtis dissimilarities showed that the individual significantly explained the greatest amount of variation in fungal community composition (PERMANOVA: *p* = 0.003, *R*^2^ = 0.28), with no significant associations between fungal community composition and our three sampling methods (PERMANOVA: *p* = 0.36, *R*^2^ = 0.38; Supplementary Table 6).

Following the collection of fecal samples, we performed shotgun sequencing on a second subset of samples. Mucosal brushes were excluded from the second sample set because a pilot shotgun sequencing run revealed these samples contained a large proportion of human-derived reads (Supplementary Fig. 2). Based on estimates of Shannon diversity and species richness, the microbiomes in fecal samples were significantly more diverse than those in the mucosal aspirates (LME: *p* = 0.007 and *p* = 0.002, respectively) and marginally more diverse than those in lavage aspirates (LME: *p* = 0.053 and *p* = 0.047, respectively; Fig. 2e). Visualization of sample beta diversities revealed a cluster of fecal samples that partially overlapped with mucosal and lavage aspirates (Fig. 2f). PERMANOVA showed that the individual explained the greatest amount of variation in microbiome composition (PERMANOVA: *p* = 0.001, *R*^2^ = 0.72; Supplementary Table 7). In comparison, the sampling method explained 15% of the variation in the microbiome (PERMANOVA: *p* = 0.001). Fecal samples had a mean relative abundance of 63% for Firmicutes, 27% for Bacteroides, 3.5% for Actinobacteria, and 4.5% for Proteobacteria. Mucosal aspirates and lavage aspirates were more similar and had a mean relative abundance of 73 and 75% for Firmicutes, 15 and 11% for Bacteroides, 4.7 and 5.2% for Actinobacteria, and 4.0 and 6.6% for Proteobacteria, respectively (Supplementary Fig. 3). Differential abundance analysis revealed 42 microbes whose abundances significantly differed between fecal samples and mucosal aspirates (ANCOM2: *p*-adj <0.05; Supplementary Table 8). Five microbes were differentially abundant between fecal samples and lavage aspirates (Supplementary Table 9), and no microbes were significantly different between mucosal aspirates and lavage aspirates (ANCOM2; *p*-adj >0.05).

### The microbiomes of polyps and healthy opposite wall tissue are similar within individuals

**Fig. 3:**
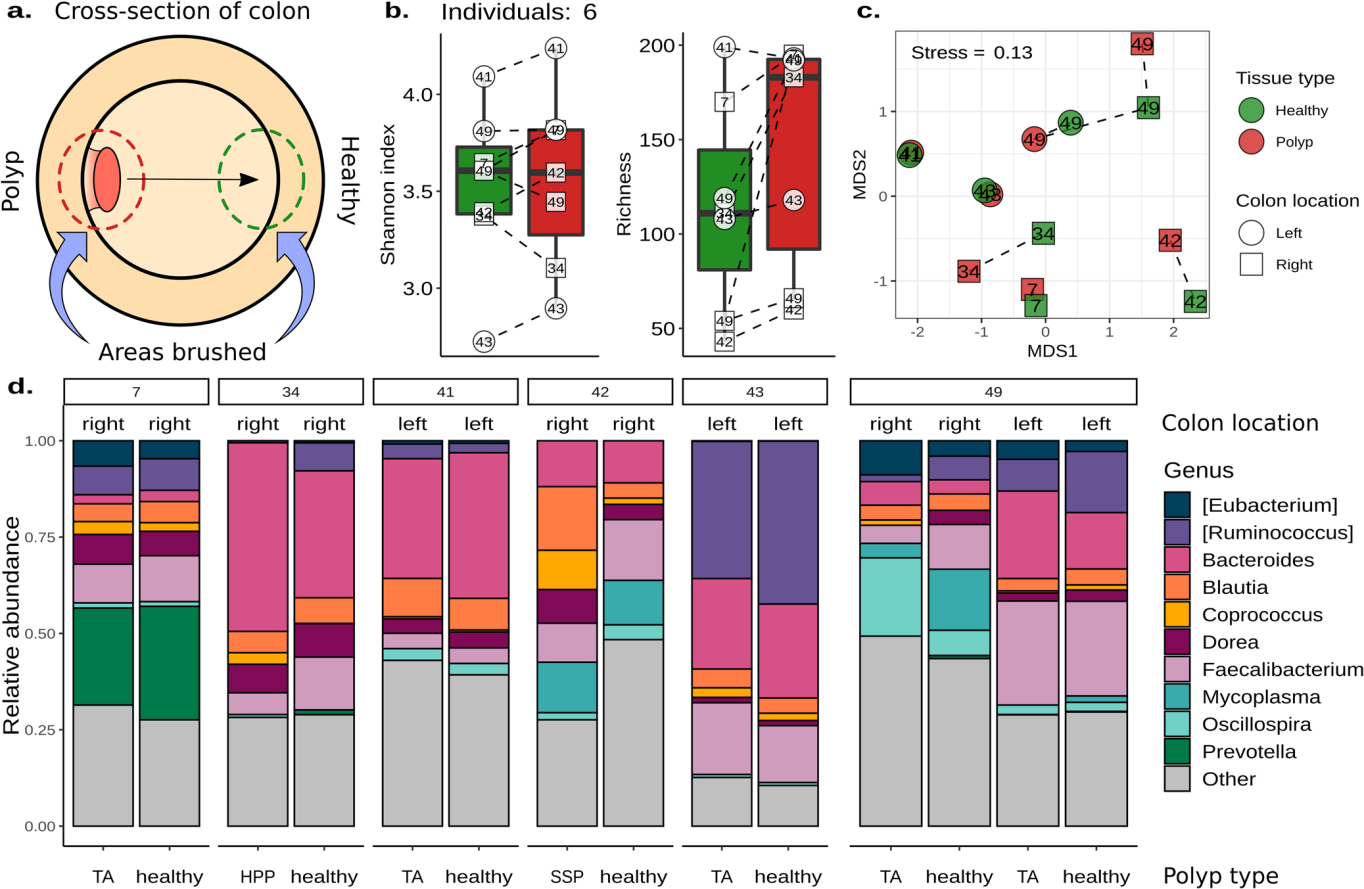
The microbiomes of polyps and healthy opposite wall tissue are similar within individuals. **a** An illustration of the sampling strategy used to characterize the microbial community of 16S mucosal brushes from polyps (red) and healthy opposite wall tissue (green). **b** Box plots of Shannon diversity and richness estimates from a polyp and healthy opposite wall brushes. The center line within each box defines the median, boxes define the upper and lower quartiles, and whiskers define 1.5x the interquartile range. **c** Non-metric multidimensional scaling of Bray–Curtis dissimilarities of a polyp and healthy opposite wall tissue brushes. Each point is one sample, with multiple samples per individual. The individual of origin is denoted numerically within each point. The shape of each point denotes the right (proximal) and left (distal) sides of the colon. **d** The relative abundance of the top ten microbial genera across all samples. Samples are grouped by each individual and labeled by polyp type, where TA tubular adenoma, HPP hyperplastic polyp, and SSP sessile serrated polyp. Source data for Fig. 3b–d are provided as a Source Data file.

To characterize the microenvironment of polyps, 14 mucosal brush samples from six subjects were collected from polyps and healthy opposite wall tissue and sequenced as part of the first sample set (Fig. 3a). Based on 16S sequencing, we observed no significant differences in Shannon diversity or richness between polyp and healthy opposite wall tissue from within the same individual (Fig. 3b). We did observe significantly increased richness in samples from the left-sided colon when compared to the right-sided colon (Fig. 3b, LME: *p* = 0.01). With respect to beta diversity, there were no significant differences across polyp and healthy opposite wall tissue pairs (PERMANOVA: *p* = 0.87, *R*^2^ = 0.18; Fig. 3c and Supplementary Table 10). We were unable to identify any differentially abundant microbes between polyp and opposite wall tissue brushes. Microbiomes were mostly individualistic, with subject origin explaining 55% of the variance in microbiome composition (PERMANOVA: *p* = 0.02; Fig. 3d and Supplementary Table 10).

### Tubular adenoma-bearing, serrated polyp-bearing, and polyp-free individuals have distinct microbiomes

We next reanalyzed all samples from the first and second sample sets to examine whether the subject’s polyp type of a sample (polyp-free vs. tubular adenoma-bearing vs. serrated polyp-bearing) was significantly associated with microbial diversity and composition. In both 16S and shotgun data, we observed no significant differences between subject types based on Shannon diversity or richness estimates (LME: *p* > 0.05; Supplementary Fig. 4). In ITS data, we observed significantly increased Shannon diversity, but not richness, in samples from polyp-free individuals when compared to those from TA-bearing individuals (LME: *p* = 0.03; Supplementary Fig. 4). Beta diversity analysis of 16S and ITS data from the first sample set demonstrated that subject type explained 4 and 2% of the variance associated with the microbiome, respectively (16S PERMANOVA: *p* = 0.001; Supplementary Table 5 and ITS PERMANOVA: *p* = 0.204; Supplementary Table 6).

Within the second sample set, we found significant associations between the microbiome and subject type, explaining 2% of the observed variance (PERMANOVA: *p* = 0.001; Supplementary Table 7). This association was examined further by testing each pairwise subject type comparison within each sample type. Between TA vs. SP-bearing mucosal aspirates, subject type significantly explained 2.7% of the variance associated with the microbiome (PERMANOVA: *p* = 0.001; Supplementary Table 11). The proportion of significant variance associated with subject type was reduced to 1.9% for polyp-free vs. TA-bearing mucosal aspirates (PERMANOVA: *p* = 0.001) and 1.5% for polyp-free vs. SP-bearing mucosal aspirates (PERMANOVA: *p* = 0.001; Supplementary Table 11). An association between microbiome composition and subject type was not observed when testing lavage aspirates (PERMANOVA: *p* = 0.47; Supplementary Table 12) or fecal samples (PERMANOVA: *p* = 0.10; Supplement Table 13) alone.

We then performed an in-depth investigation of each subject type’s microbiome using only the second sample set of mucosal aspirates due to their larger comparable sample size. Differential abundance analysis demonstrated that *Eggerthella lenta* was significantly depleted in SP-bearing aspirates when compared to polyp-free ones (Kruskal–Wallis, KW: *p*-adj = 0.032). *E. lenta* also demonstrated a lower abundance in SP-mucosal aspirates when compared to TA aspirates, but this decrease was not significant (KW: *p*-adj = 0.099). Supplementary Fig. 5 suggest that *E. lenta* was also depleted in 16S mucosal aspirates, but this result was not statistically significant either.

**Fig. 4:**
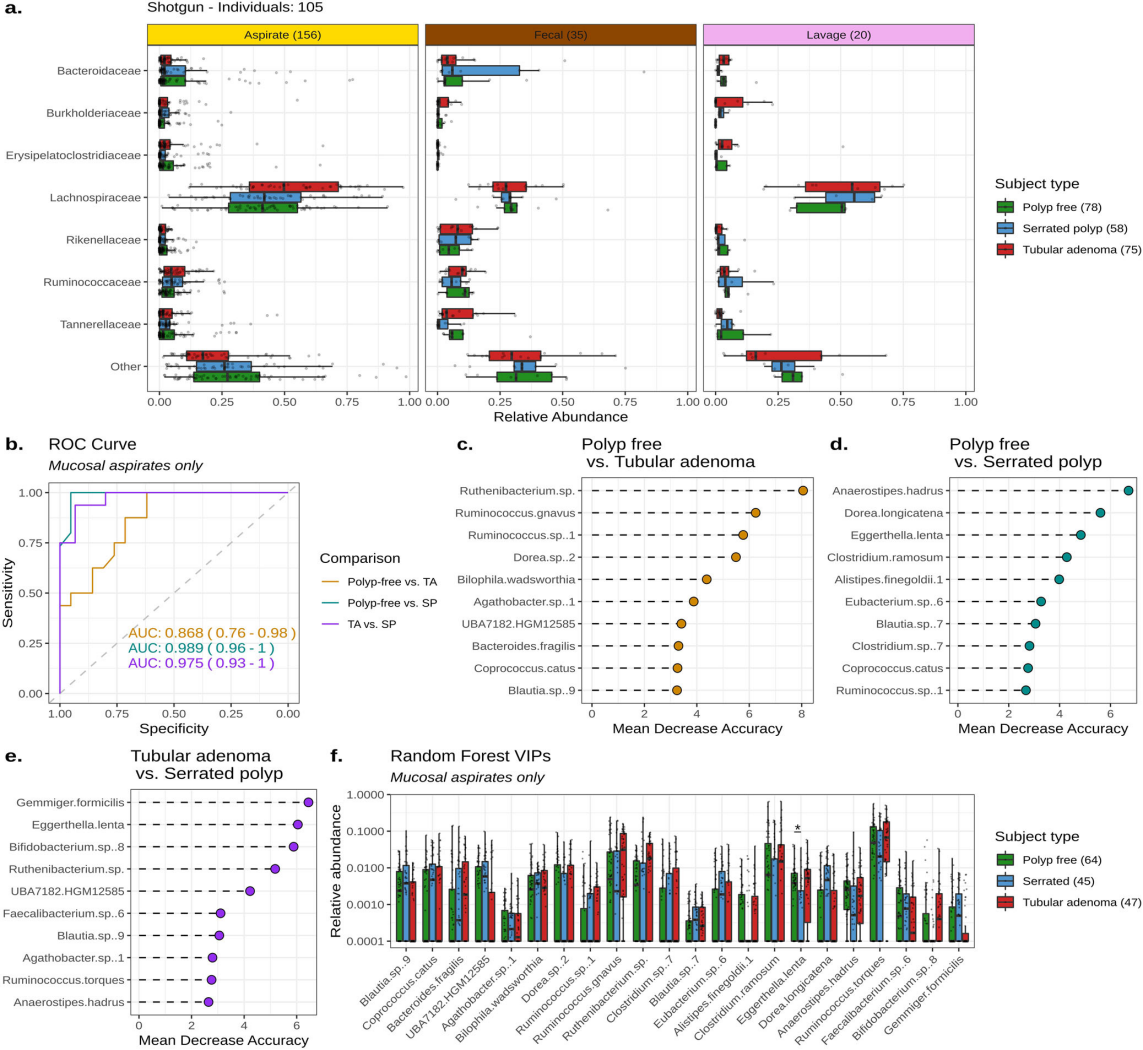
Tubular adenoma-bearing, serrated polyp-bearing, and polyp-free individuals have distinct microbiomes. **a** Box plots of the top seven most abundant microbial families across all samples from Sample set 2 shotgun data. The number of samples per sampling method and subject type are denoted parenthetically, with multiple samples per individual. **b** A receiver operating characteristic (ROC) curve illustrating the true positive rate (Sensitivity, y-axis) versus the false positive rate (Specificity, x-axis) produced by Random Forest classification of Sample set 2 mucosal aspirates. The area under the curve (AUC) value for each Random Forest is displayed with a 90% confidence interval. **c–e** The top ten variables of importance for each pairwise Random Forest classification. Variables are sorted by their mean decrease in accuracy, with larger means contributing greater to Random Forest performance. **f** Box plots displaying the relative abundances of the top Random Forest variables of importance. Each point is one sample, with multiple samples per individual. A pseudo-count of 0.0001 was added to visualize samples which had relative abundances of zero since the y-axis is scaled to log_10_. The center line within each box defines the median, boxes define the upper and lower quartiles, and whiskers define 1.5x the interquartile range. Significant comparisons (Kruskal–Wallis: *p*-adj <0.05) are denoted with an asterisk (*). Source data are provided as a Source Data file.

Despite few differentially abundant microbes, taxonomic visualization suggested that TA-bearing mucosal aspirates were distinct compared to polyp-free and SP-bearing mucosal aspirates (Fig. 4a and Supplementary Fig. 6). Therefore, we examined if microbial composition could be used to predict the subject type origin of mucosal aspirates. Random Forest (RF) classified mucosal aspirates from each pairwise subject type comparison with moderate to high accuracy, producing area under curve (AUC) values of 0.87–0.99 (Fig. 4b). The top variables of importance for the classification of polyp-free versus TA-bearing mucosal aspirates were *Ruthenibacterium* sp., *Ruminococcus gnavus*, *Ruminococcus* sp.*, Dorea* sp., and *Blautia* sp. (Fig. 4c). For polyp-free versus SP-bearing RF classification, *Anaerostipes hadrus*, *Dorea longicatena*, *E. lenta, Clostridium ramosum*, and *Alistipes finegoldii* were the most important variables (Fig. 4d). Lastly, *Gemmiger formicilis*, *E. lenta, Bifidobacterium* sp., *Ruthenibacterium* sp., and UBA7182 HGM12585 were the top microbes of importance for the SP-bearing versus TA-bearing RF classification (Fig. 4e). Figure 4f displays the relative abundances for the top variables of importance in all RF comparisons.

### Microbiome functional potential is distinct across sampling methods and subject types

**Fig. 5:**
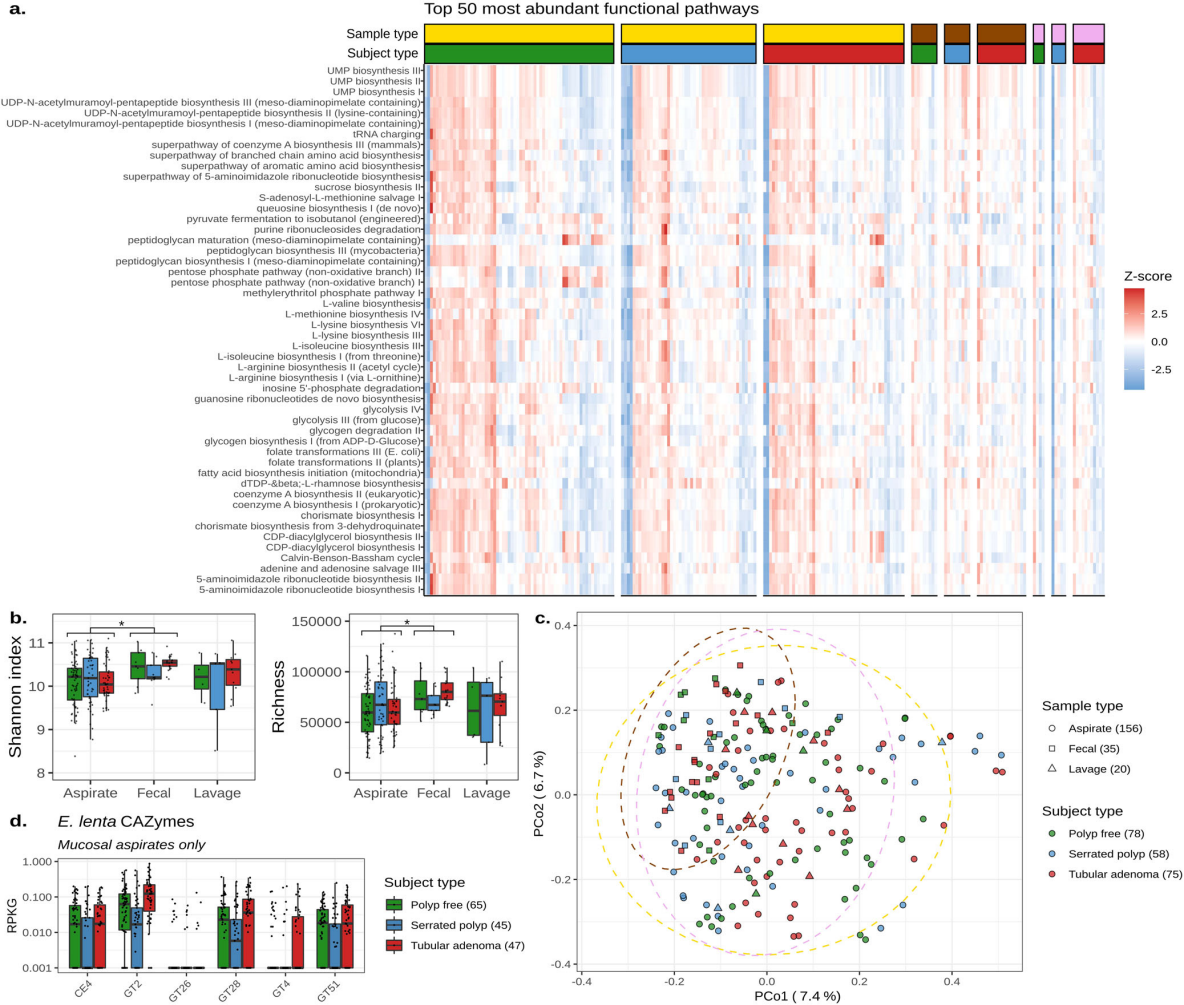
Microbiome functional potential is distinct across sampling methods and subject types. **a** A heatmap displaying the *z*-scores of the top 50 most abundant microbial pathways found within the second sample set. Each column is one sample, with multiple samples per individual. Samples are clustered by sample and subject types. Yellow represents mucosal aspirates, brown represents fecal samples, and purple represents lavage aspirates. Within-subject types, green represents polyp-free samples, blue represents serrated polyp samples, and red represents tubular adenoma samples. **b** Box plots showing the Shannon diversity and richness of individual microbial genes across the second sample set mucosal aspirates, lavage aspirates, and fecal samples. Significant comparisons (Linear-mixed effects: *p* < 0.05) are denoted with an asterisk (*). **c** Principal coordinate analysis of per-gene Bray–Curtis dissimilarities. Each point represents one sample. Ellipses are drawn to represent the 95% confidence interval of each sample type’s distribution. The number of samples per sampling method and subject type are annotated parenthetically. **d** Box plots showing the abundance of *E. lenta*-specific carbohydrate-active enzymes in reads per kilobase per genome equivalent. Only mucosal aspirates from the second sample set are shown, with the number of mucosal aspirates per subject type being denoted parenthetically. The center line within each box defines the median, boxes define the upper and lower quartiles, and whiskers define 1.5x the interquartile range. Source data are provided as a Source Data file.

The functional characteristics of our shotgun metagenomes were next explored. Pathway analysis resulted in the discovery of 507 metabolic pathways, which were generally conserved across subject types (Fig. 5a and Supplementary Fig. 7). As a result, we did not identify any differentially abundant pathways (KW: *p*-adj >0.05). Additionally, pairwise RF classification of functional pathways resulted in lower AUC values when compared to taxonomic RF classification (Supplementary Figs. 8, 9).

Subsequently, we analyzed individual microbial genes, whose composition exhibited a higher correlation to microbial taxonomy (Mantel: *p* = 0.001, *r* = 0.70) when compared to functional pathways (Mantel: *p* = 0.001, *r* = 0.33). Like previous taxonomic results, we found that fecal samples had significantly increased Shannon diversity (LME: *p* = 0.034) and gene richness estimates (LME: *p* = 0.021) when compared to mucosal aspirates, but not mucosal lavages (Fig. 5b). Principal coordinate analysis resulted in fecal samples clustering together, with no obvious clustering based on subject type (Fig. 5c). This was supported by PERMANOVA, which confirmed an association between functional metagenome and sampling method, explaining 10.8% of the observed variance (PERMANOVA: *p* = 0.001; Supplementary Table 14). By comparison, the individual of origin explained ~76% of the observed variance in the functional microbiome (PERMANOVA: *p* = 0.001; Supplementary Table 14) and the subject type explained 1.3% of the observed variance (PERMANOVA: *p* = 0.001; Supplementary Table 14).

We concluded our analysis by searching for differentially abundant genes among subject types using mucosal aspirates but did not find any after adjusting for the false discovery rate (KW: *p*-adj >0.05). Supplementary Fig. 10 demonstrates that the majority of the genes determined to be differentially abundant before FDR correction originated from the class *Coriobacteriia*, which *E. lenta* belongs to. Given that *E. lenta* metabolizes plant lignans in the gut and was found to be depleted in SP-bearing mucosal aspirates, we decided to examine which *E. lenta*-specific carbohydrate-active enzymes (CAZymes) were present in our metagenomes. We found six CAZymes, of which four had decreased abundance in SP-mucosal aspirates. These were a carbohydrate esterase, family 2 (CT2), and three glycosyl transferases from families 2, 28, and 51 (GT2, GT28, and GT51; Fig. 5d). A complete list of differentially abundant genes, their functions, and taxonomy before FDR correction can be found in the supplement (Supplementary File 1).

## Discussion

In this study, we used direct and indirect methods to sample the colon and characterize the microbiomes of polyp-free and polyp-bearing individuals. Using amplicon sequencing, we found that microbiomes of mucosal brushes and mucosal aspirates did not significantly differ in diversity or composition. In contrast, the microbiomes of fecal samples were significantly more diverse and compositionally distinct when compared to those from mucosal aspirates.

Due to their ease of collection, fecal samples are frequently used to study the human microbiome in the context of CRC. However, fecal samples poorly represent the microbiota adherent to the colon mucosa and instead capture those found in the intestinal lumen^20,21^. Their increased diversity and paucity of mucosa-associated microbes suggest that fecal samples are less ideal for studying premalignant polyps, which have fewer pronounced signatures of microbial dysbiosis when compared to carcinomas. This is supported by Peters et al., who found greater compositional changes in the microbiomes of fecal samples from advanced conventional adenomas when compared to those from non-advanced adenomas^17^. The decreased sensitivity of fecal samples to detect CRC-associated microbes was also highlighted by their results demonstrating significant associations between the gut microbiome and distal conventional adenoma cases, but not proximal^17^. This is also likely why Peters et al. did not observe substantial differences in the microbial compositions of HPP, SSP, and healthy samples, as serrated polyps predominantly develop in the proximal colon.

Here, we report significant associations between the gut microbiome and mucosal aspirates obtained from both the proximal and distal colon. We also observed significant differences when comparing the microbiomes of polyp-free samples to SP-bearing ones using mucosal aspirates. No such differences were seen in fecal samples, but this result may be driven by a smaller sample size. Nevertheless, these data suggest that mucosal samples are sensitive enough to study the microbiome of colorectal polyps found within the proximal colon. This contradicts a study published by Yoon et al., who found no significant compositional differences among the mucosa-associated gut microbiomes of polyp-free, TA, SSP, and CRC-bearing individuals^18^. The authors note, however, that their result was likely influenced by the small sample size, with only six samples per group and 24 samples total.

Compared to mucosal brushes, mucosal aspirates had a lower risk of damaging the intestinal epithelium, provided larger collection volumes for downstream sample processing, and resulted in lower proportions of human-derived reads during shotgun sequencing. Both methods also had similar microbiome profiles. One caveat of our approach, however, is that we did not collect mucosal aspirates from polyp tissue directly, only from healthy tissue near polyps. Therefore, it is unclear whether the three differentially abundant microbes observed between mucosal brushes and aspirates was due to the sampling method used or the tissue site (Supplementary Fig. 1). Certainly, more research is needed to further evaluate each sampling method, but we believe the advantages of mucosal aspiration outweigh the risk of mucosal brushing and any minor discrepancies in microbiome diversity and composition.

With respect to characterizing the hyperlocal microbiome of polyps and opposite colon wall tissue, mucosal brushing revealed no differences. One factor which could have disrupted any potential hyperlocal differences in the gut microbiota is the colonoscopy preparation and lavage. As part of the preparation, individuals were advised to adhere to low fiber, clear liquid diet 24 h prior to colonoscopy. Dietary fiber is important for maintaining the longitudinal and lateral organization of the microbiota within the colon, as giving mice a low fiber diet has been shown to disrupt the microbial organization of their guts^20^. Additionally, changes in diet can rapidly shift the composition of the gut microbiome, often within 24 h^7,22,23^. Another factor which could have potentially obscured the hyperlocal organization of colon epithelium further was the mechanical displacement caused by the laxative-based cleansing and colonoscopy rinse. Nevertheless, significant compositional differences between the microbiomes of samples taken from the proximal and distal colon were observed, suggesting that broad microbial organization remained present in the gut after colonoscopy preparation and lavage. It is important to note that these claims are based on data from 14 samples from six individuals, therefore, additional studies with more samples are needed to validate the reproducibility of our findings.

Comparatively, compositional differences were observed in the gut microbiome across TA-bearing, SP-bearing, and polyp-free individuals using mucosal sampling. Notably, we demonstrated that the microbial composition of each subject type was distinct enough to accurately predict the origin of mucosal aspirates using RF. These findings suggest that the gut microbiome plays different roles in the adenoma-carcinoma sequence and the serrated pathway. In the adenoma-carcinoma sequence, the gut microbiome exists in, and potentially contributes to, an inflammatory environment to promote colorectal carcinogenesis.

Data obtained from the second set of mucosal aspirates supports that TA-bearing subjects had an altered microbiome composition associated with inflammation and CRC development. These samples trended towards a higher abundance of *Lachnospiraceae*, such as *Ruminococcus gnavus*, which has been previously associated with CRC and inflammatory bowel disease, and *C. scindens*, which can metabolize excess primary bile acids not absorbed by the small intestine into secondary bile acids (Supplementary Fig. 6)^24–26^. High concentrations of secondary bile acids can cause host oxidative stress, nitrosative stress, DNA damage, apoptosis, and mutations^27^. Secondary bile acids also act as farnesoid X receptor antagonists, resulting in enhanced *wnt* signaling in the adenoma-carcinoma sequence^28^. RF classification also identified *Bacteroides fragilis* as a top variable of importance, which was elevated in TA mucosal aspirates. *B. fragilis* produces a metalloprotease that causes oxidative DNA damage and cleaves the tumor suppressor protein, E-cadherin^29–31^.

Unlike the adenoma-carcinoma sequence, the microbiome in the serrated pathway remains understudied. *Fusobacterium nucleatum*, which has been implicated in the adenoma-carcinoma sequence because of its ability to activate *wnt* signaling, has also been described as having a role in serrated CRC development^32^. *F. nucleatum* abundance is associated with serrated pathway lesions and features, such as mismatch repair deficiency, MLH1 methylation, CpG island methylator phenotype, and high microsatellite instability^14^. Here, we did not find differences in *F. nucleatum* abundances across HPPs, SSPs, TAs, or polyp-free controls. Instead, we most prominently found that *E. Lenta* and its CAZymes were depleted in mucosal aspirates from SP-bearing individuals, a result that spanned 16S and shotgun data.

*E. lenta* metabolizes inert plant lignans in the gut into bioactive enterolignans, such as enterolactone and enterodiol^33^. These enterolignans have anti-proliferative and anti-inflammatory effects and help modulate estrogen signaling, lipid metabolism, and bile acid regulation^34^. They have also been associated with reduced cancer risk^35^. Diets rich in plant fiber have been associated with decreased CRC risk^6,36^. Fiber is fermented by the intestinal microbiota to produce short-chain fatty acids, including acetate, butyrate, and propionate. Butyrate is the primary energy source for colonocytes and has anti-inflammatory and anti-tumor properties^37–39^. Butyrate also is involved in the epigenetic expression of genes as a histone deacetylase inhibitor^40^. In the serrated pathway, the gene SLC5A8, which mediates short-chain fatty acid uptake into colonic epithelial cells, is frequently inhibited via promoter methylation, suggesting that dietary fiber may be required for proper cellular epigenetic regulation^41^.

Further evidence of dietary fiber potentially playing a role in the serrated pathway was the identification of *A. hadrus* as the most important variable in differentiating polyp-free vs SP-bearing mucosal aspirates by RF. *A. hadrus* is a butyrate-producing microbe and was depleted in SP-bearing mucosal aspirates^42^. Taken together, we hypothesize that low dietary fiber consumption facilitates aberrant epigenetic modifications within colonocytes to promote serrated polyp development, but studies which combine both mucosal sampling methods and dietary information are needed to test this hypothesis.

In conclusion, the complex and individualistic nature of the human gut microbiome has made it difficult to mechanistically link the microbiome with colorectal carcinogenesis. By describing the association between the gut microbiota and two colorectal polyp types with several sampling methods, our study provides insight into potential mechanisms for the epigenetic-based serrated pathway of CRC. In addition, our data underscores the importance of distinguishing between different pathways of colorectal carcinogenesis when investigating the gut microbiome. Finally, transitioning future microbiome studies to mucosal sampling methods may enable the discovery of previously unassociated CRC microbes.

## Methods

### Subject recruitment and criteria

Individuals who presented for colonoscopy with indications of screening for, or a prior history of, colorectal polyps were asked to participate in the study. Written and informed consent was obtained from each subject and was required for participation. Subjects who were pregnant and had taken antibiotics within 6 weeks of colonoscopy or with known inflammatory bowel diseases were excluded. In total, 140 individuals were recruited for this study. Of the 140 individuals, 50 were found to be polyp-free, 45 had one or more TAs, 33 had polyps originating from the serrated pathway (HPPs or SSPs), and 12 had unknown or other pathologies.

### Colonoscopy preparation, procedure, and sample collection

Before a colonoscopy, subjects were asked to adhere to a clear liquid diet for 24 h. Bowel cleansing was done using Miralax, or polyethylene glycol with electrolytes administered as a split dose, 12 and 5 h before the procedure. Sample collection focused on two direct and two indirect microbiome sampling methods (Fig. 1). The first direct sampling method involved brushing the mucosa of the colon during colonoscopy (Method #1 in Fig. 1). Brushing was performed on suspected polyps and on opposing healthy colon tissue to compare their microenvironments. Since mucosal brushes can potentially damage or agitate the intestine, we also employed a method of direct microbiome sampling in which colonoscopy washing fluid was sprayed directly onto the target mucosa and immediately re-suctioned into a storage vial (Method #2 in Fig. 1). Participants with suspected polyps had mucosal washing aspirates taken on healthy tissue near the polyp, but no mucosal aspirates were taken from polyps directly. The first indirect sampling method involved collecting an aspirate of the post-colonoscopy lavage fluid (Method #3 in Fig. 1). This lavage fluid was produced from rinsing the wall of the colon throughout the procedure and was collected in a container outside the subject. All samples were collected in sterile cryogenic tubes and placed on ice until the colonoscopy procedure was finished. Afterward, the samples were stored at −80 °C. Additional information collected included indication for the procedure, age, sex, ethnicity, BMI, family history, and findings, including the size, shape, location, and pathology of all polyps sampled.

### Patient-directed collection of fecal samples

For the second indirect microbiome sampling method, subjects were encouraged to send follow-up fecal samples four to six weeks post-colonoscopy (Method #4 in Fig. 1). Subjects were provided with a fecal collection kit, which contained collection equipment, prepaid shipping labels, and Zymo DNA/RNA shield preservation buffer (R1101). Subjects who complied were compensated $20 USD. Samples were returned via the United States Postal Service. After arrival, samples were stored at −80 °C. Thirty-eight fecal samples were returned, bringing our total number of samples collected to 1883. A summary of the sample types can be found in Supplementary Table 1.

### Polyp and subject type classification

Polyp biopsies collected during colonoscopy were sent to a pathologist for classification. This information was then recorded for the corresponding mucosal brush and aspirate samples. Pathology reports were also used to broadly categorize all samples collected from an individual by their polyp pathology. We referred to this as the “subject type” and the three categories were polyp-free subjects, tubular adenoma-bearing subjects (TA-bearing), and serrated polyp-bearing (SP-bearing) subjects, which included both HPPs and SSPs. For example, if a sample was taken from the healthy intestinal tissue of an individual who was found to have a TA, that sample and all others from the same individual would be included in the TA-bearing subject type. Three individuals had both a TA and an SSP and were classified as SP-bearing subjects.

### DNA extraction

Two separate DNA extractions were performed in this study, yielding two different sample sets (Table 1). Sample set 1 DNA extractions included mucosal brushes, mucosal aspirates, and lavage aspirates only. Sample set 2 DNA extractions occurred later and included mucosal aspirates, lavage aspirates, and fecal samples. All samples were thawed on ice for DNA extraction. For mucosal aspirates and lavage aspirate samples, 250 uL of fluid were taken from each sample and then DNA was extracted using ZymoBiomics DNA Miniprep Kit (D4300) according to the manufacturer’s protocol. For mucosal brushes, 750 uL of ZymoBIOMICS Lysis Solution was mixed with the brushes in their original sterile cryogenic tube and vortexed for 5 min to suspend the contents of the brush into the solution. The solution was then transferred and extracted according to the manufacturer’s protocol. Fecal samples stored in the Zymo DNA/RNA shield were thawed, mixed by vortexing, and 750 uL of the fecal plus buffer mix was extracted according to the manufacturer’s protocol.

### 16S amplicon library preparation and sequencing

Samples from the first set underwent 16S and ITS amplicon sequencing. We targeted the V4 region of the bacterial 16S rRNA gene using the 515 F and 926 R primers. For each sample, the V4 region was amplified using 25 uL polymerase chain reaction (PCR) volumes with the following reagents: 12.5 uL of 1x AccustartII PCR tough mix (QuantaBio 95142), 9.5 uL of PCR grade water, 1 uL of 10 mg/mL BSA, 0.5 ng of extracted genomic DNA, and 0.5 uL of 0.2 uM 515 F, and 926 R primers each. The 515 F primer contained the Illumina adapter sequence and barcode. Each sample was amplified using a thermocycler for 30 cycles (94 °C for 3 min; 94 °C for 45 s, 55 °C for 30 s, 72 °C for 20 s; repeat steps 2–4 30 times; 72 °C for 10 min). The resultant amplicons were quantified using the Qubit dsDNA HS Assay Kit (Life technologies Q32851) according to the manufacturer’s protocol and pooled at equimolar concentrations. The pooled amplicon library was cleaned and concentrated using Agencourt AMPure XP beads (Beckman–Coulter A63880) according to the manufacturer’s protocol. Equimolar PhiX was added at 10% final volume to the amplicon library and sequenced on the Illumina MiSeq platform, yielding 300 bp paired-end sequences. A total of 200 samples with an average of 41,578 ± 35,920 (σ) reads per sample were obtained for 16S amplicons.

### ITS amplicon library preparation and sequencing

Fungi from the first sample set were characterized by targeting the ITS2 region of the 18S rRNA gene for amplification. We used the ITS9f and ITS4r primers, as described by Looby et al.^43^. PCR was performed in 25 uL volumes, consisting of: 12.5 uL of 1x AccustartII PCR tough mix, 9.5 uL of PCR grade water, 1 uL of 10 mg/mL BSA, 0.5 ng of extracted genomic DNA, and 0.5 uL of 0.3 uM ITS9f and barcoded ITS4r primers each. Amplification was performed with the following thermocycler settings: 94 °C for 5 min, 35 cycles of 95 °C for 45 s, 50 °C for 1 min, 72 °C for 90 s, and a final extension step of 72 °C for 10 min. Afterward, we quantified, pooled, and cleaned our ITS2 amplicons using the same methods as our 16S amplicons. Our ITS2 library was combined with our 16S library and sequenced simultaneously in the reverse complementary orientation. This yielded 150 samples with an average of 22,252 ± 17,000 (σ) ITS reads per sample.

### Shotgun library preparation and sequencing

The second sample set was sequenced using shotgun sequencing. Libraries were prepared using the Illumina DNA prep kit (20018705), using our low-volume protocol^44^. Briefly, a maximum of 5 uL or 50 ng (whichever was reached first) of DNA from each sample was tagmented with 2 uL of tagmentation master mix for 15 min at 55 °C. Afterward, 1 uL of tagmentation stop buffer was added to each sample and incubated at 37 °C for 15 min. The samples were washed with the provided buffer according to the manufacturer’s protocol, then PCR was performed with 12.5 uL reaction volumes with the following reagents: 6.25 uL of KAPA HiFi HotStart ReadyMix (Roche Life Science KK2602), 2.75 uL of PCR grade water, 1.25 uL of 1 uM i5 and i7 index adapters each, and 0.5 uL of 10 uM forward and reverse KAPA HiFi polymerase primers each. PCR amplification was done with the settings: 72 °C for 3 min, 98 °C for 3 min, 12 cycles of 98 °C for 45 s, 62 °C for 30 s, 72 °C for 2 min, and a final extension step of 72 °C for 1 min. Samples were pooled and size selection was performed per the manufacturer’s protocol. Libraries were packaged on dry ice and shipped overnight to Novogene Corporation Inc. (Sacramento, CA) to be sequenced using Illumina’s Hiseq 4000 for 150 bp paired-end sequencing. This yielded 257 samples with an average of 1,267,359 ± 690,384 (σ) reads per sample.

### Taxonomic assignment of sequencing data

For the first sample set, 16S and ITS amplicon sequences were processed using Qiime2-2019.1^45^. Demultiplexing was performed using the “q2-demux” function with the “emp-paired” preset. Sequencing reads were quality filtered, had chimeric sequences, PhiX, and singletons removed, and were clustered into amplicon sequence variants (ASVs) using the “q2-dada2” function with the default parameters plus trunc_len_f = 280, trunc_len_r = 220, trim_left_f = 5, and trim_left_r = 5^46^. This reduced the average number of reads per sample to 30,051 ± 24,768 (σ) for 16S amplicons and 3517 ± 9154 (σ) for ITS amplicons. Taxonomic assignment of 16S and ITS reads was done using the “classify-sklearn” function in Qiime2 with the default parameters. The databases used for classification were the Greengenes database (Version 13.8) for 16S data and the UNITE database (Version 8.0) for ITS data^47,48^. This produced 182 samples with an average of 28,343 ± 23,150 (σ) high-quality, taxonomically assigned reads per sample for 16S amplicons and 131 samples with an average of 3461 ± 8357 (σ) high-quality, taxonomically assigned reads per sample for ITS amplicons.

For the second sample set shotgun data, we first removed sequencing adapters using the “bbduk.sh” script from BBMap v38.79 with the default parameters^49^. Next, we demultiplexed our samples using “demuxbyname.sh” script from BBMap using the default parameters. After demultiplexing, sequences were quality filtered using PRINSEQ + + v1.2 with the parameters trim_left = 5, trim_right = 5, min_len = 100, trim_qual_right = 28, and min_qual_mean = 25^50^. This yielded an average of 1,209,001 ± 643,544 (σ) high-quality reads per sample. Removal of human-derived reads was performed with Bowtie2 v2.3.5.1 on default settings by removing reads which aligned to the reference human genome, hg38^51^. This resulted in 257 samples with an average of 1,102,247 ± 643,325 (σ) high-quality, non-human reads per sample. Lastly, we used IGGSearch v1.0 on the “lenient” preset (-min-reads-gene = 1 –min-perc-genes = 15 –min-sp-quality = 25) to assign operational taxonomic units (OTU) to our quality-filtered sequences^52^. This produced 238 samples with an average of 24,888 ± 16,340 (σ) high-quality marker gene reads per sample.

### Taxonomic analysis

Data analysis was performed using R v3.6.3. For all sequencing runs, a synthetic microbial community DNA standard (ZymoBIOMICS D6305) was included as a control. When necessary, the first step in our compositional analysis was filtering taxa from all samples that contaminated the community standard control. Next, unassigned and mitochondrial reads were removed from our samples. Afterward, we excluded 16S and ITS samples with fewer than 2500 and 1000 reads, respectively, as these samples did not have sufficient read depth to fully represent their microbial diversity (Supplementary Fig. 11). Filtering was not required, nor performed for shotgun samples. The final number of 16S, ITS, and shotgun samples with high-quality, taxonomically assigned reads was 147, 98, and 238, respectively (Supplementary Tables 2–4).

The alpha diversities for both amplicon and shotgun data were obtained using the “diversity” and “specnumber” functions from the Vegan v2.5-6 package, using the default parameters. Linear-mixed effect models (LME) were used for significance testing among alpha diversities to account for random effects, such as plate batching effects, and multiple measurements per individual using the nlme package, v3.1-148. For all datasets, beta diversities were obtained using the “adonis” function in Vegan to generate Bray–Curtis distance matrices and perform PERMANOVA significance testing from compositional data. Beta diversity was visualized using non-metric multidimensional scaling (NMDS) ordination obtained from the ‘metaMDS’ function in Vegan. Matrix correlation was assessed using the ‘mantel’ function in Vegan.

### Differential abundance testing

Our primary focus with the first sample set was to compare the microbial compositions of different sample types within the same individual. Therefore, we used ANCOM v2.1 in R to test for differentially abundant microbes since it can account for multiple variables and random effects^53^. We used ANCOM with “sample type” as our variable of interest (mucosal brushes vs. mucosal aspirates vs. colonoscopy lavage aspirates) and the individual of origin as a random effect. Other parameters included “*p*_adjust = FDR” to control for the false discovery rate, and significance was determined at <0.05.

For shotgun data, our primary focus was to compare the microbial composition of different subject types (Polyp-free vs. TA-bearing vs. SP-bearing). We used a univariate Kruskal–Wallis (KW) test with independent hypotheses weighting (IHW). IHW increases power while controlling the false discovery weight by utilizing covariate data that are independent of the null hypothesis^54^. Before testing, we excluded samples with “Unknown/Other” subject types and filtered taxa that were not present in at least one-third of the samples. We also eliminated repeated measurements by averaging the microbial relative abundances of left and right mucosal aspirates from the same individual. Kruskal–Wallis tests were performed for each taxon with the subject type as the variable. The IHW v1.14.0 package was used to correct *p* values for the false discovery rate, using the sum of read counts per taxon across all samples as our covariate. FDR-adjusted *p* values < 0.05 were considered significant. When visualizing relative abundances using a log_10_ scale, a pseudo-count of 0.0001 was added to prevent the removal of samples containing zeroes.

### Random Forests

Random Forests (RF) were performed on shotgun-sequenced mucosal aspirates to determine if the subject type of a sample could be predicted based on microbial composition. To do this, we used the rfPermute v1.9.3 package in R. We began by filtering taxa which were not present in at least one-third of mucosal aspirate samples. Two-thirds of the 156 shotgun mucosal aspirates were used for training the RF classifiers, while the remaining one-third was used for testing our RF models. RfPermute parameters were set to importance = TRUE, nrep = 100, ntree = 501, and mtry = 8. Afterward, we generated receiver-operator curves (ROC) using the “roc” function with default settings (pROC v1.18.0 package). Variables of importance were visualized with the ‘VarImpPlot’ function in the rfPermute package.

### Pathway enrichment analysis

Pathway enrichment analysis was done using unassembled shotgun reads with HUMAnN v3.0.1^55^. The program was run using the default parameters and the ChocoPhlAn v296 and UniRef90 v201901b databases were used for alignment. The “humann_renorm_table” and “humann_join_tables” functions were used to create a pathway abundance matrix of normalized counts in copies per million. Significantly enriched pathways between subject types were determined with a Kruskal–Wallis test using IHW. The false discovery rate was corrected by using the total sum of normalized counts per pathway as our covariate. Significance was determined at FDR <0.05. *Z*-scores were calculated from pathway abundances and then were visualized on a heatmap generated by the “dist” and “hclust” functions in R.

### Functional metagenomic analysis

Analysis of individual microbial genes was performed by cross-assembling reads into contiguous sequences using MEGAHIT v1.1.1^56^. Contigs smaller than 2500 bp were discarded and the remainder had open reading frames (ORFs) identified by Prodigal v2.6.3^57^. The resulting ORFs were functionally annotated using eggNOG mapper v2.0, using the eggNOG v5.0 database^58^. Individual samples were aligned to annotated ORFs using Bowtie2 v2.3 to obtain per sample ORF abundances. Per sample, ORF abundances were compiled into a single ORF abundance table using the “pileup.sh” script from BBMap. ORF counts were normalized to reads per kilobase per genome equivalent using MicrobeCensus v1.1.1 on default settings^59^. Principal coordinate analysis was performed using the “cmdscale” function from Vegan to visualize the functional metagenome composition among sample and subject types. PERMANOVA and differential abundance testing were performed in the same manner as with taxonomy.

### Reporting summary

Further information on research design is available in the Nature Research Reporting Summary linked to this article.

## Supplementary information

Supplementary Figures and Tables

Differentially abundant genes

ASV/OTU tables

Reporting Summary

## Data Availability

Sequencing data is available on the Sequence Read Archive under the BioProject ID, PRJNA745329. The source data used to generate Figs. 2, 3b–d, 4, and 5 are provided as a Source Data File. The ASV/OTU tables and corresponding metadata used to generate the source data can be found in “Supplementary_File 2.xlsx”. Larger files used in our functional metagenomic analysis are available on the Dryad Digital Repository (10.7280/D1J10M). Additional data and materials are available upon reasonable request.

## Source data

Source Data File

## Code availability

All code for data processing and analysis are available on GitHub at: https://github.com/Javelarb/ACS_polyp_study.
