# Supplementary material for: Distinct colon mucosa microbiomes associated with tubular adenomas and serrated polyps
Source: NPJ Biofilms Microbiomes. 2022 Aug 29;8:69. doi: 10.1038/s41522-022-00328-6 (PMC9424272; doi:10.1038/s41522-022-00328-6)
Supplement: Supplementary file 1 — Supplementary Figures and Tables [file 41522_2022_328_MOESM1_ESM.pdf]

Supplementary Tables:

	Polyp-free	TA-bearing	SP-bearing (HPP/SSP)	Unknown or Other	Total
**Mucosal brushes [On-polyp]**	197 [0]	168 [58]	112 (61 [18]/ 51 [17])	48 [14]	525
**Mucosal aspirates**	350	280	195 (111/84)	72	897
**Lavage aspirates**	159	135	93 (54/39)	36	423
**Fecal samples**	9	17	9 (6/3)	3	38
**Total**	715	600	409	159	1883

Supplementary Table 1: A table showing the number of samples collected. Across rows, the number of each sample type is listed. For mucosal brushes, the number within the bracket corresponds to the number of brush samples taken directly from polyp tissue (as opposed to brushing non-polyp tissue). Across columns, the subject type classification is given. The number of samples per hyperplastic polyps (HPP) and sessile serrated polyps (SSP) are denoted parenthetically for the SP-bearing category. Samples were collected from a total of 140 unique individuals.

16S	Polyp-free	TA-bearing	SP-bearing (HPP/SSP)	Unknown or Other	Total
**Mucosal brushes [On-polyp]**	12 [0]	34 [11]	18 (8 [2]/10 [4])	0 [0]	64
**Mucosal aspirates**	17	24	11 (5/7)	0	52
**Lavage aspirates**	10	13	8 (3/5)	0	31
**Fecal samples**	0	0	0	0	0
**Total**	39	71	37	0	147

Supplementary Table 2: A table showing the number of samples with high quality sequencing reads in sample set 1, using 16S amplicon sequencing. Across rows, the number of each sample type is listed. For mucosal brushes, the number within the bracket corresponds to the number of brush samples taken directly from polyp tissue (as opposed to brushing non-polyp tissue). Across columns, the subject type classification is given. The number of samples per hyperplastic polyps (HPP) and sessile serrated polyps (SSP) are denoted parenthetically for the SP-bearing category. A total of 38 unique individuals were represented in this data.

ITS	Polyp-free	TA-bearing	SP-bearing (HPP/SSP)	Unknown or Other	Total
**Mucosal brushes [On-polyp]**	12 [0]	20 [7]	9 (1 [0]/8 [1])	0	41
**Mucosal aspirates**	13	17	11 (2/9)	0	41
**Lavage aspirates**	7	4	5 (1/4)	0	16
**Fecal samples**	0	0	0	0	0
**Total**	32	41	25	0	98

Supplementary Table 3: A table showing the number of samples with high quality sequencing reads in sample set 1, using ITS amplicon sequencing. Across rows, the number of each sample type is listed. For mucosal brushes, the number within the bracket corresponds to the number of brush samples taken directly from polyp tissue (as opposed to brushing non-polyp tissue). Across columns, the subject type classification is given. The number of samples per hyperplastic polyps (HPP) and sessile serrated polyps (SSP) are denoted parenthetically for the SP-bearing category. A total of 34 unique individuals were represented in this data.

WGS	Polyp-free	TA-bearing	SP-bearing (HPP/SSP)	Unknown or Other	Total
**Mucosal brushes [On-polyp]**	0	0	0	0	0
**Mucosal aspirates**	64	47	45 (24/17)	23	179
**Lavage aspirates**	5	11	4 (2/2)	1	21
**Fecal samples**	9	17	9 (6/3)	3	38
**Total**	78	75	58	27	238

Supplementary Table 4: A table showing the number of samples with high quality sequencing reads in sample set 2, using whole-genome shotgun sequencing. The number of each sample type is listed across rows. Across columns, the subject type classification is given. Additionally, the number of samples per hyperplastic polyps (HPP) and sessile serrated polyps (SSP) are denoted parenthetically for the SP-bearing category. A total of 117 unique individuals were represented in this data.

FACTOR	DoF	SoS	MS	F MODEL	R^2	P-VAL
**BMI**	1	1.41	1.40	9.83	0.03	0.001
**AGE**	1	0.98	0.98	6.88	0.02	0.001
**ETHNICITY**	4	5.18	1.29	9.07	0.10	0.001
**SEX**	1	1.29	1.29	9.00	0.02	0.001
**SUBJECT TYPE**	2	2.26	1.13	7.92	0.04	0.001
**SUBJECT TYPE: INDIVIDUAL**	28	27.15	0.97	6.79	0.51	0.001
**SUBJECT TYPE: INDIVIDUAL: SAMPLE TYPE**	63	7.96	0.13	0.88	0.15	0.992
**RESIDUALS**	46	6.57	0.14		0.13	
**TOTAL**	146	50.46			1.00	

PERMANOVA formula: 16S_ASV_table ~ BMI + Age + Ethnicity + Sex + Subject Type / Individual / Sample Type, strata = Plate

Supplementary Table 5: PERMANOVA analysis of brushes, mucosal aspirates, and lavage aspirates from the first sample set using 16S sequencing. The distance matrix method used was Bray-Curtis dissimilarity. Subject type includes polyp-free, tubular adenoma-bearing, and serrated polyp-bearing samples. Individuals are nested within subject type, and sample type is nested within the individual.

FACTOR	DoF	SoS	MS	F MODEL	R^2	P-VAL
**BMI**	1	0.53	0.53	1.26	0.01	0.066
**AGE**	1	0.43	0.43	1.03	0.01	0.341
**SEX**	1	0.44	0.44	1.05	0.01	0.297
**ETHNICITY**	3	1.40	0.47	1.12	0.03	0.108
**SUBJECT TYPE**	2	0.91	0.46	1.10	0.02	0.204
**SUBJECT TYPE: INDIVIDUAL**	24	11.77	0.49	1.17	0.28	0.003
**SUBJECT TYPE: INDIVIDUAL: SAMPLE TYPE**	38	16.03	0.42	1.01	0.38	0.361
**RESIDUALS**	25	10.43	0.42		0.26	
**TOTAL**	95	41.93			1.00	

PERMANOVA formula: ITS_ASV_table ~ BMI + Age + Sex + Ethnicity + Subject Type / Individual / Sample Type

Supplementary Table 6: PERMANOVA analysis of brushes, mucosal aspirates, and lavage aspirates from the first sample set using ITS sequencing. The distance matrix method used was Bray-Curtis dissimilarity. Subject type includes polyp-free, tubular adenoma-bearing, and serrated polyp-bearing samples. Individuals are nested within subject type, and sample type is nested within the individual.

FACTOR	DoF	SoS	MS	F MODEL	R^2	P-VAL
**BMI**	1	0.72	0.72	10.24	0.01	0.001
**AGE**	1	0.67	0.67	9.45	0.01	0.001
**ETHNICITY**	4	2.66	0.70	9.39	0.04	0.001
**SEX**	1	0.71	0.71	10.02	0.01	0.001
**SUBJECT TYPE**	2	1.43	0.72	10.07	0.02	0.001
**SUBJECT TYPE: INDIVIDUAL**	86	53.79	0.63	8.83	0.72	0.001
**SUBJECT TYPE: INDIVIDUAL: SAMPLE TYPE**	37	10.97	0.30	4.19	0.15	0.001
**RESIDUALS**	56	3.96	0.07		0.04	
**TOTAL**	188	74.94			1.00	

PERMANOVA formula: OTU_table ~ BMI + Age + Sex + Ethnicity + Subject Type / Individual / Sample Type, strata = Plate

Supplementary Table 7: PERMANOVA analysis of mucosal aspirates, lavage aspirates, and fecal samples from the second sample set using shotgun sequencing. The distance matrix method used was Bray-Curtis dissimilarity. Subject type includes polyp-free, tubular adenoma-bearing, and serrated polyp-bearing samples. Individuals are nested within subject type, and sample type is nested within the individual.

taxa_id	W	detected_0.9	detected_0.8	detected_0.7	detected_0.6
**UBA1381.sp.**	149	TRUE	TRUE	TRUE	TRUE
**Ruminococcus.torques**	147	TRUE	TRUE	TRUE	TRUE
**Clostridium.ramosum**	146	TRUE	TRUE	TRUE	TRUE
**D16.sp..2**	146	TRUE	TRUE	TRUE	TRUE
**Ruminococcus.gnavus**	145	TRUE	TRUE	TRUE	TRUE
**Oscillibacter.sp.**	145	TRUE	TRUE	TRUE	TRUE
**Bacteroides.fragilis**	143	TRUE	TRUE	TRUE	TRUE
**Lachnospiraceae.sp..12**	141	TRUE	TRUE	TRUE	TRUE
**Dorea.formicigenerans**	141	TRUE	TRUE	TRUE	TRUE
**DTU089.HGM12760**	141	TRUE	TRUE	TRUE	TRUE
**D16.sp.**	141	TRUE	TRUE	TRUE	TRUE
**Ruminococcus.bicirculans**	141	TRUE	TRUE	TRUE	TRUE
**Clostridium.leptum**	139	TRUE	TRUE	TRUE	TRUE
**Eubacterium.HGM12316**	136	FALSE	TRUE	TRUE	TRUE
**Roseburia.intestinalis**	136	FALSE	TRUE	TRUE	TRUE
**Alistipes.sp..3**	135	FALSE	TRUE	TRUE	TRUE
**Coprococcus.catus**	133	FALSE	TRUE	TRUE	TRUE
**Ruminococcus.lactaris**	131	FALSE	TRUE	TRUE	TRUE
**Dorea.sp..2**	130	FALSE	TRUE	TRUE	TRUE
**Lachnospira.sp..2**	130	FALSE	TRUE	TRUE	TRUE
**Eggerthella.lenta**	129	FALSE	TRUE	TRUE	TRUE
**Flavonifractor.plautii**	128	FALSE	TRUE	TRUE	TRUE
**Eubacterium.sp..9**	127	FALSE	TRUE	TRUE	TRUE
**Lachnospira.pectinoschiza**	124	FALSE	TRUE	TRUE	TRUE
**Tyzzerella.sp..1**	123	FALSE	FALSE	TRUE	TRUE
**Faecalibacterium.HGM13278**	123	FALSE	FALSE	TRUE	TRUE
**Eubacterium.sp..6**	121	FALSE	FALSE	TRUE	TRUE
**Escherichia.coli**	121	FALSE	FALSE	TRUE	TRUE
**Lachnospiraceae.HGM11862**	120	FALSE	FALSE	TRUE	TRUE
**Erysipelatoclostridium.sp..2**	119	FALSE	FALSE	TRUE	TRUE
**Eubacterium.sp..15**	119	FALSE	FALSE	TRUE	TRUE
**Clostridium.bartlettii**	119	FALSE	FALSE	TRUE	TRUE
**Coprococcus.comes**	116	FALSE	FALSE	TRUE	TRUE
**DTU089.HGM12731**	116	FALSE	FALSE	TRUE	TRUE
**Faecalibacterium.sp..6**	116	FALSE	FALSE	TRUE	TRUE
**Bilophila.wadsworthia**	115	FALSE	FALSE	TRUE	TRUE
**Parabacteroides.distasonis**	114	FALSE	FALSE	TRUE	TRUE
**Intestinimonas.butyriciproducens**	114	FALSE	FALSE	TRUE	TRUE
**ER4.sp.**	112	FALSE	FALSE	TRUE	TRUE
**Dorea.longicatena.1**	111	FALSE	FALSE	TRUE	TRUE
**Clostridium.glycyrrhizinilyticum**	111	FALSE	FALSE	TRUE	TRUE
**UBA7182.HGM12585**	109	FALSE	FALSE	TRUE	TRUE

Supplementary Table 8: Table of differentially abundant OTUs across fecal samples and mucosal aspirates from the second sample set using shotgun sequencing. Significance testing was performed using ANCOM2 (FDR <

0.05), adjusting for repeated measurements. “Detected 0.7” means that the microbe was differentially abundant in 70% of comparisons, which is the minimum for a microbe to be considered differentially abundant between categories.

taxa_id	W	detected_0.9	detected_0.8	detected_0.7	detected_0.6
**Oscillibacter.sp.**	95	TRUE	TRUE	TRUE	TRUE
**Lachnospiraceae.sp..12**	94	FALSE	TRUE	TRUE	TRUE
**Ruminococcus.torques**	84	FALSE	FALSE	TRUE	TRUE
**Dorea.formicigenerans**	83	FALSE	FALSE	TRUE	TRUE
**Ruminococcus.bicirculans**	76	FALSE	FALSE	TRUE	TRUE

Supplementary Table 9: Table of differentially abundant OTUs across fecal samples and lavage aspirates from the second sample set using shotgun sequencing. Significance testing was performed using ANCOM2 (FDR < 0.05), adjusting for repeated measurements. "Detected 0.7" means that the microbe was differentially abundant in 70% of comparisons, which is the minimum for a microbe to be considered differentially abundant between categories.

FACTOR	DoF	SoS	MS	F MODEL	R^2	P-VAL
**COLON LOCATION**	1	0.74	0.74	3.19	0.15	0.015
**SUBJECT TYPE**	1	0.42	0.42	1.83	0.08	0.124
**SUBJECT TYPE: INDIVIDUAL**	4	2.77	0.69	3.00	0.55	0.020
**SUBJECT TYPE: INDIVIDUAL: TISSUE SITE**	6	0.92	0.15	0.66	0.18	0.875
**RESIDUALS**	1	0.23	0.23		0.04	
**TOTAL**	13	5.07			1.00	

PERMANOVA formula: 16S_brushes_ASV_table ~ Colon location + Subject type / Individual / Tissue site

Supplementary Table 10: PERMANOVA analysis of brushes from the first sample set using 16S sequencing. The distance matrix method used was Bray-Curtis dissimilarity. Subject type includes tubular adenoma-bearing and serrated polyp-bearing samples. Tissue site includes polyp and healthy opposite wall brushes. Individuals are nested within subject type, and tissue site is nested within the individual.

A. Polyp-free vs. tubular adenoma mucosal aspirates

Factor	DoF	SoS	MS	F MODEL	R2	P-VAL
**BMI**	1	0.932049	0.932049	14.28858	0.02497	0.001
**Age**	1	0.510753	0.510753	7.829994	0.013683	0.001
**Ethnicity**	3	2.093075	0.697692	10.69581	0.056075	0.001
**Sex**	1	0.826017	0.826017	12.66306	0.02213	0.001
**Colon Location**	1	0.096297	0.096297	1.476259	0.00258	0.04
**Prep Type**	2	1.129164	0.564582	8.6552	0.030251	0.001
**Subject Type**	1	0.731753	0.731753	11.21797	0.019604	0.001
**Subject Type: Patient**	48	28.46318	0.592983	9.090592	0.762551	0.001
**Residuals**	39	2.543985	0.06523		0.068155	
**Total**	97	37.32627			1	

B. Polyp-free vs. serrated polyp mucosal aspirates

Factor	DoF	SoS	MS	F MODEL	R2	P-VAL
**BMI**	1	0.514723	0.514723	7.280071	0.014123	0.001
**Age**	1	0.610964	0.610964	8.641267	0.016763	0.001
**Ethnicity**	3	2.448353	0.816118	11.5429	0.067176	0.001
**Sex**	1	0.669934	0.669934	9.475329	0.018381	0.001
**Colon Location**	1	0.159277	0.159277	2.252762	0.00437	0.003
**Prep Type**	2	1.39528	0.69764	9.867187	0.038283	0.001
**Subject Type**	1	0.572898	0.572898	8.102883	0.015719	0.001
**Subject Type: Patient**	44	27.38873	0.622471	8.804025	0.75147	0.001
**Residuals**	38	2.686714	0.070703		0.073716	
**Total**	92	36.44687			1	

C. Tubular adenoma vs. serrated polyp mucosal aspirates

Factor	DoF	SoS	MS	F MODEL	R2	P-VAL
**BMI**	1	0.785529	0.785529	10.16161	0.023108	0.001
**Age**	1	0.848928	0.848928	10.98174	0.024973	0.001
**Ethnicity**	3	1.881841	0.62728	8.114507	0.055357	0.001
**Sex**	1	0.690819	0.690819	8.936442	0.020322	0.001
**Colon Location**	1	0.143375	0.143375	1.854707	0.004218	0.009
**Prep Type**	2	1.163965	0.581982	7.528535	0.03424	0.001
**Subject Type**	1	0.932052	0.932052	12.05705	0.027418	0.001
**Subject Type: Patient**	46	25.07423	0.545092	7.05132	0.737598	0.001
**Residuals**	32	2.473713	0.077304		0.072768	
**Total**	88	33.99446			1	

PERMANOVA formulas: Aspirate_OTU_table ~ BMI + Age + Ethnicity + Sex + Colon Location +Prep Type + Subject Type / Patient, strata = Plate

Supplementary Table 11: Pairwise PERMANOVA analysis of lavage aspirates from the second sample set using shotgun sequencing. The distance matrix method used was Bray-Curtis dissimilarity. Prep type refers to the

laxative used during colonoscopy prep. Table 11A compares polyp-free and tubular adenoma subject types, table 11B compares polyp-free and serrated polyp subject types, and table 11C compares tubular adenoma and serrated polyp subject types.

FACTOR	DoF	SoS	MS	F MODEL	R^2	P-VAL
**SUBJECT TYPE**	2	0.86	0.43	1.20	0.14	0.114
**PREP TYPE**	2	0.82	0.41	1.15	0.13	0.151
**AGE**	1	0.46	0.46	1.30	0.07	0.074
**BMI**	1	0.41	0.41	1.16	0.07	0.310
**SEX**	1	0.35	0.35	0.98	0.06	0.553
**EHTNICITY**	2	0.86	0.43	1.21	0.14	0.081
**RESIDUALS**	7	2.50	0.36		0.39	
**TOTAL**	16	6.27			1.00	

PERMANOVA formula: Lavage_OTU_table ~ Subject type + Prep type + Age + BMI + Sex + Ethnicity, strata = Plate

Supplementary Table 12: PERMANOVA analysis of lavage aspirates from the second sample set using shotgun sequencing. The distance matrix method used was Bray-Curtis dissimilarity. Subject type includes polyp-free, tubular adenoma-bearing and serrated polyp-bearing samples. Prep type refers to the laxative used during colonoscopy prep.

FACTOR	DoF	SoS	MS	F MODEL	R^2	P-VAL
**SUBJECT TYPE**	2	0.83	0.42	1.11	0.07	0.168
**AGE**	1	0.40	0.40	1.07	0.03	0.309
**BMI**	1	0.43	0.43	1.13	0.04	0.201
**SEX**	1	0.38	0.38	1.03	0.03	0.402
**ETHNICITY**	3	1.03	0.34	0.92	0.09	0.778
**RESIDUALS**	23	8.62	0.37		0.74	
**TOTAL**	31	11.70			1.00	

PERMANOVA formula: Fecal_OTU_table ~ Subject type + Age + BMI + Sex + Ethnicity, strata = Plate

Supplementary Table 13: PERMANOVA analysis of fecal samples from the second sample set using shotgun sequencing. The distance matrix method used was Bray-Curtis dissimilarity. Subject type includes polyp-free, tubular adenoma-bearing and serrated polyp-bearing samples. Prep type refers to the solution used during colonoscopy prep.

FACTOR	DoF	SoS	MS	F MODEL	R^2	P-VAL
**BMI**	1	0.004	0.004	19.36	0.015	0.001
**AGE**	1	0.002	0.002	8.76	0.007	0.001
**ETHNICITY**	4	0.013	0.003	14.26	0.043	0.001
**SEX**	1	0.001	0.001	4.28	0.003	0.012
**SUBJECT TYPE**	2	0.004	0.002	8.74	0.013	0.001
**SUBJECT TYPE: INDIVIDUAL**	86	0.221	0.003	11.73	0.768	0.001
**SUBJECT TYPE: INDIVIDUAL: SAMPLE TYPE**	38	0.031	0.001	3.72	0.108	0.001
**RESIDUALS**	57	0.013	0.001		0.043	
**TOTAL**	190	0.289			1.000	

PERMANOVA formula: Gene_table ~ BMI + Age + Ethnicity + Sex + Subject type / Individual / Sample type, strata = Plate

Supplementary Table 14: PERMANOVA analysis of functional genes within mucosal aspirates, lavage aspirates, and fecal samples from the second sample set using shotgun sequencing. The distance matrix method used was Bray-Curtis dissimilarity. Subject type includes polyp-free, tubular adenoma-bearing, and serrated polyp-bearing samples. Individuals are nested within subject type, and sample type is nested within the individual.

Supplementary Figures

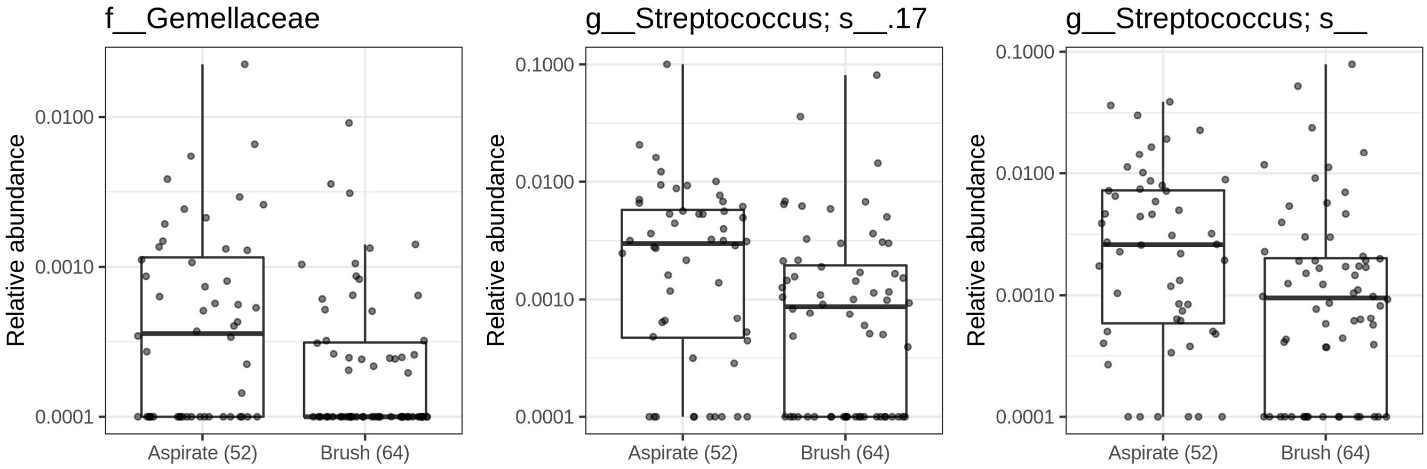

Supplementary Figure 1: Box plots displaying the relative abundance of microbes determined to be differentially abundant by ANCOM2 (p-adj < 0.05). Data is from mucosal brushes and mucosal aspirates from the first sample set. Each point is one sample, with multiple samples per individual. Plots are labeled with the most specific taxonomic rank for each ASV. A pseudo-count of 0.0001 was added to visualize samples which had relative abundances of zero, since the y axis is scaled to $\log_{10}$. The center line within each box defines the median, boxes define the upper and lower quartiles, and whiskers define 1.5x the interquartile range.

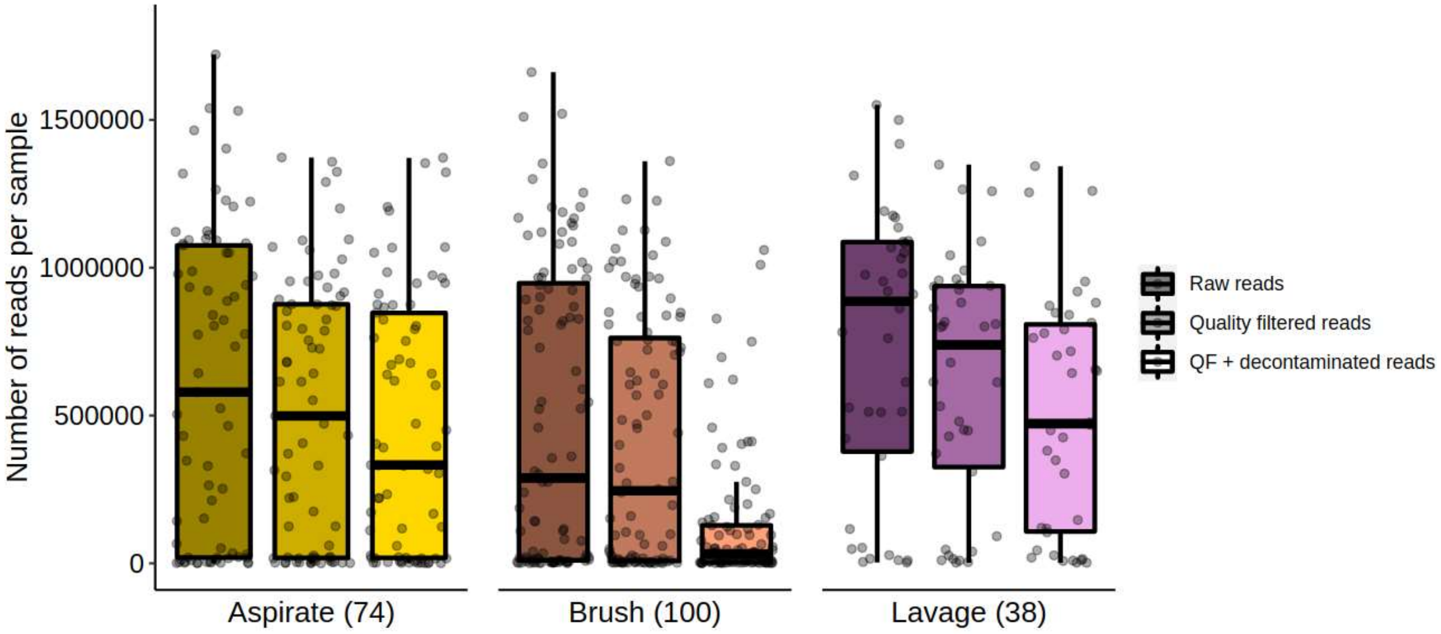

Supplementary Figure 2: Box plots showing the number of reads per sample produced by a pilot shotgun sequencing run using mucosal brushes, mucosal aspirates, and lavage aspirates from the first sample set. Each point is one sample, with multiple samples per individual. The number of samples per sampling method is denoted parenthetically. The center line within each box defines the median, boxes define the upper and lower quartiles, and whiskers define 1.5x the interquartile range. 'Raw reads' refers to the number of reads produced by the Illumina NextSeq platform. 'Quality filtered reads' refers to the number of reads after removing reads with a quality score lower than a mean of 28. 'QF + decontaminated reads' refers to the number of reads after removing human-derived reads.

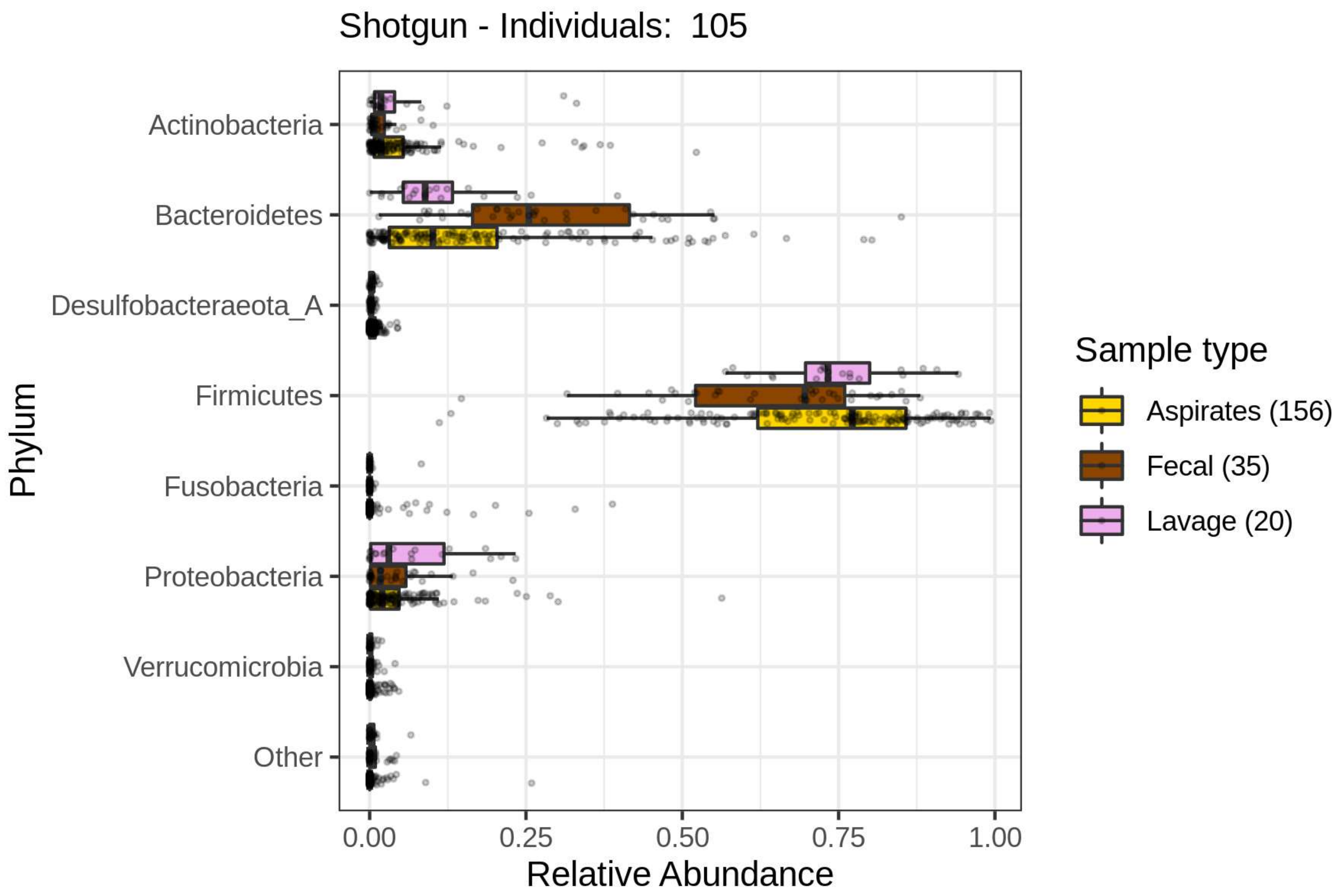

Supplementary Figure 3: Box plots showing the relative abundance of the top seven most abundant microbial phyla across mucosal aspirates, lavage aspirates, and fecal samples from the second sample set. Each point is one sample, with multiple samples per individual. The number of samples per sampling method is denoted parenthetically. The center line within each box defines the median, boxes define the upper and lower quartiles, and whiskers define 1.5x the interquartile range.

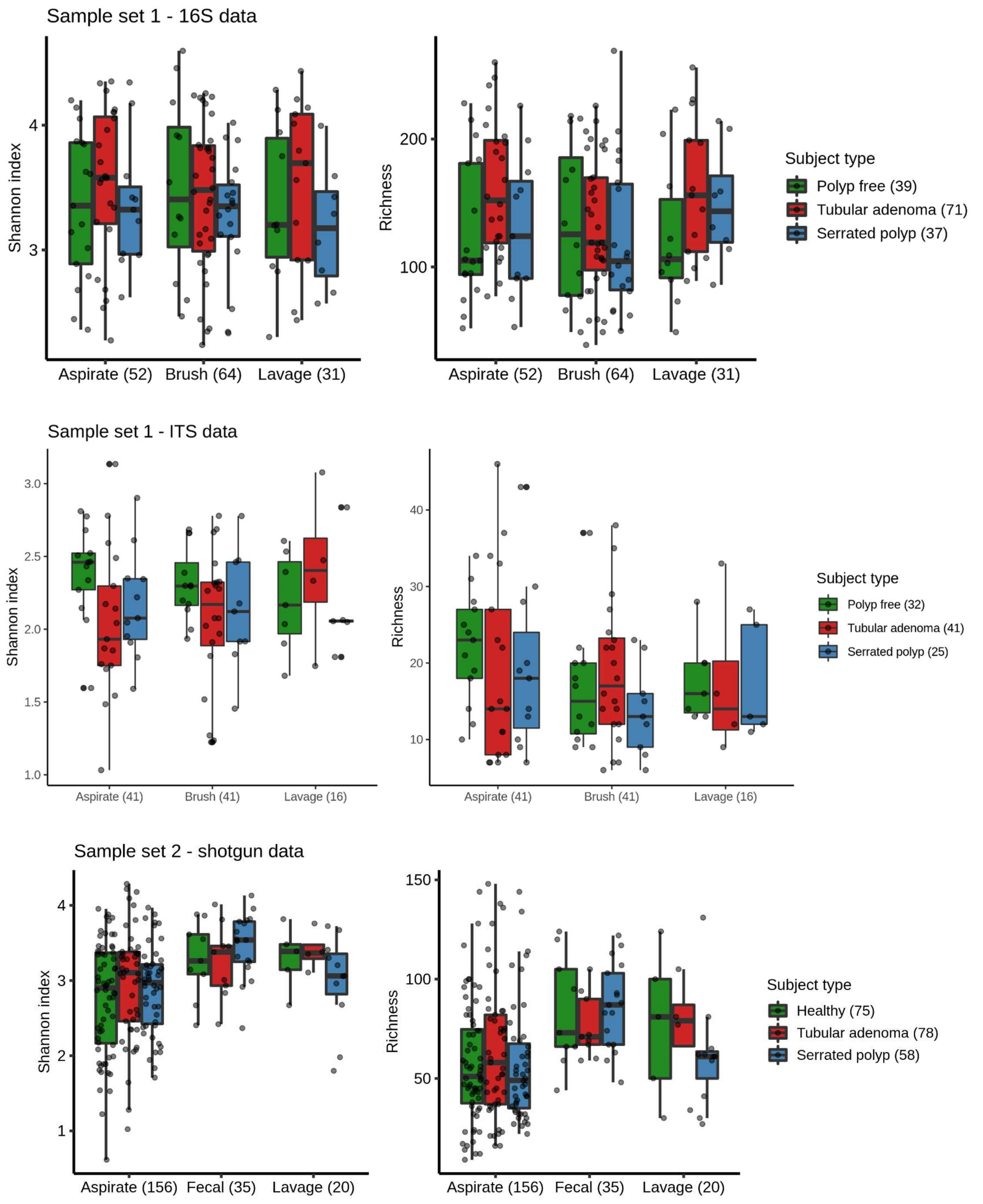

Supplementary Figure 4: Box plots showing Shannon diversity and richness estimates across the first and second sample sets. The number of samples for each sampling method and subject type are denoted parenthetically. Each

point is one sample, with multiple samples per individual. The center line within each box defines the median, boxes define the upper and lower quartiles, and whiskers define 1.5x the interquartile range. There was significantly increased Shannon diversity in polyp-free ITS samples when compared to TA samples (Linear mixed effects model: $p = 0.03$)

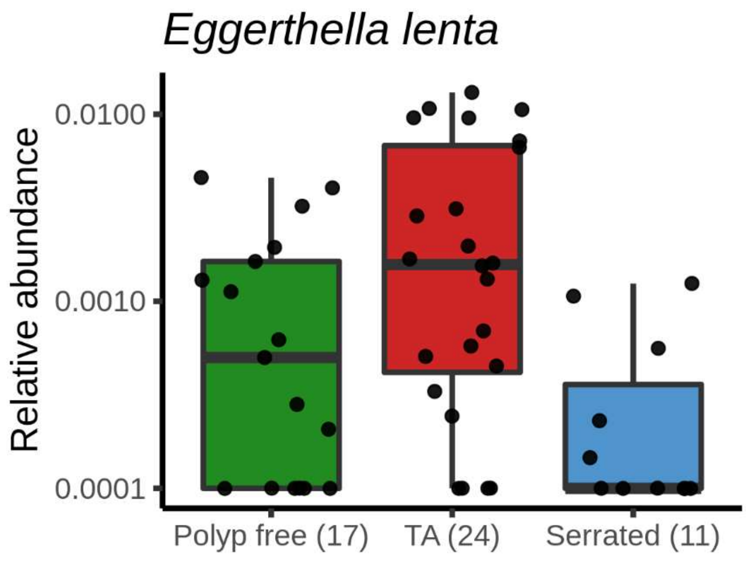

Supplementary Figure 5: A box plot showing the relative abundance of *E. lenta* in 16S mucosal aspirates from the first sample set across subject types. Each point is one sample, with multiple samples per individual. A pseudo-count of 0.0001 was added to visualize samples which had a relative abundance of zero, since the y-axis is scaled to $\log_{10}$. The center line within each box defines the median, boxes define the upper and lower quartiles, and whiskers define 1.5x the interquartile range.

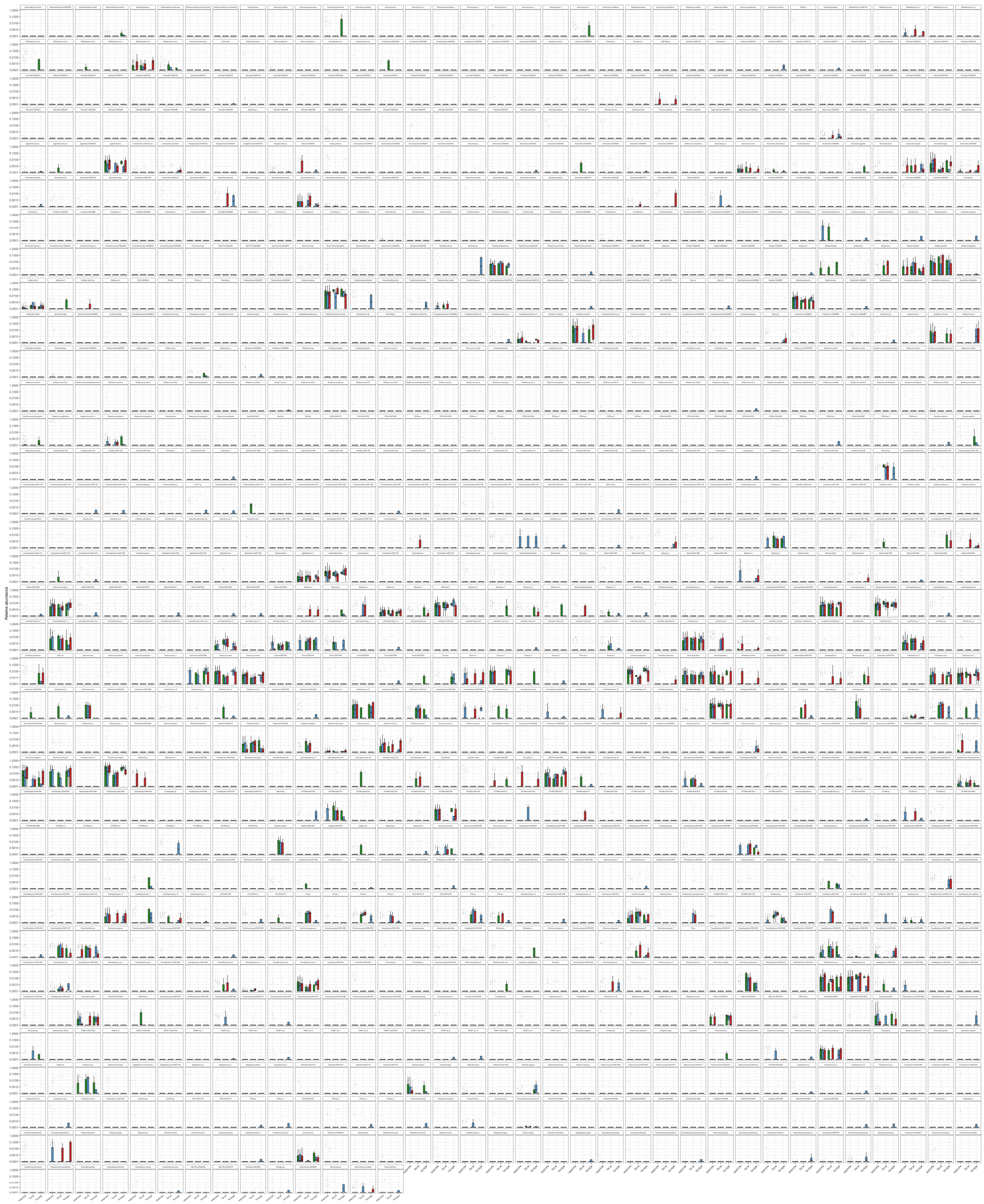

Supplementary Figure 6: Box plots showing the relative abundance of all OTUs from the second sample set.

Each point is one sample, with multiple samples per individual. Samples are faceted by sample type and colored

by subject type. Green refers to polyp-free samples, red refers to TA-bearing samples, and blue refers to SP-bearing samples. A pseudo-count of 0.0001 was added to visualize samples which had a relative abundance of zero, since the y-axis is scaled to $\log_{10}$. The center line within each box defines the median, boxes define the upper and lower quartiles, and whiskers define 1.5x the interquartile range.

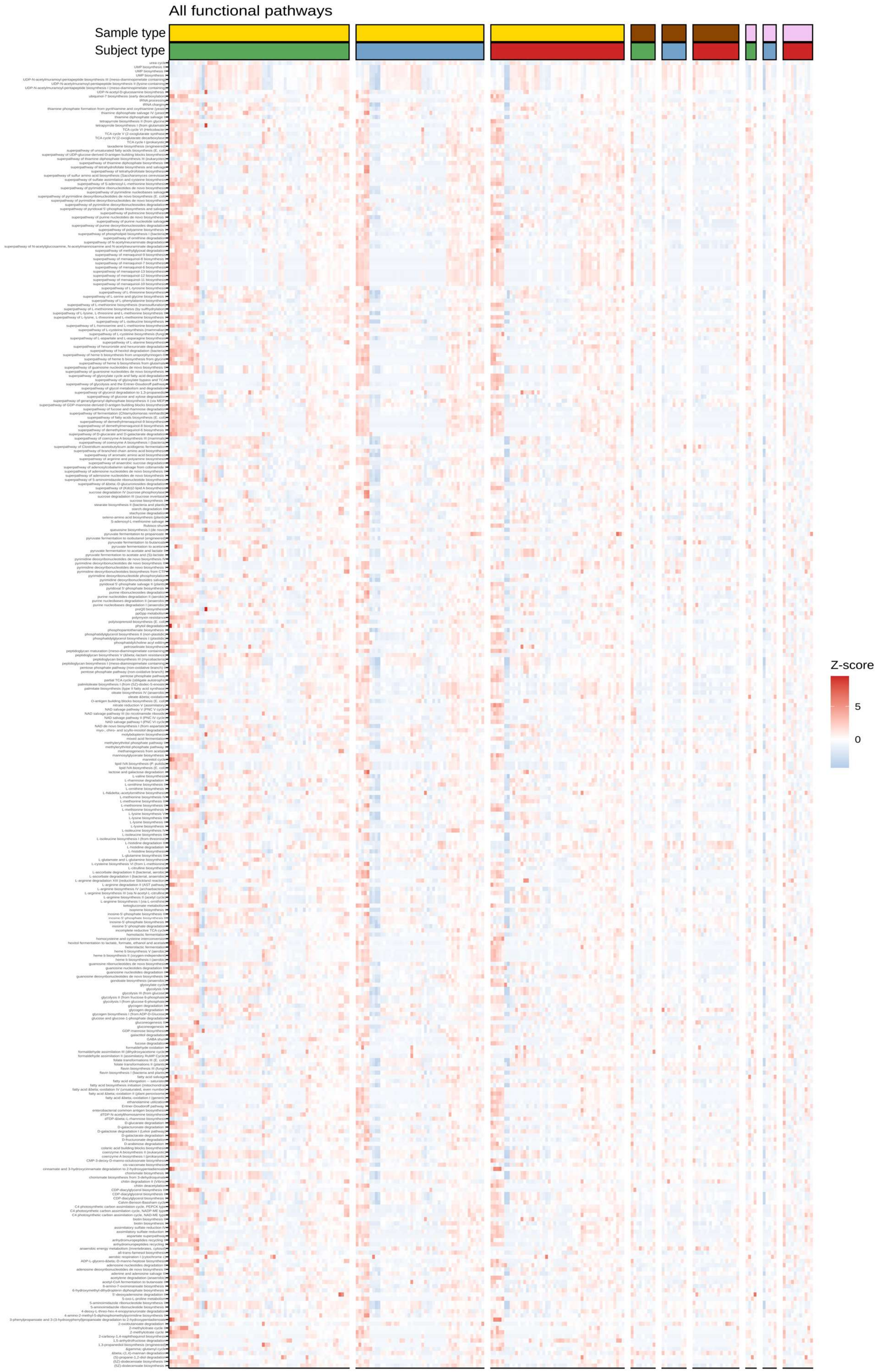
All functional pathways
Sample type
Subject type
Z-score
5
0

Supplementary Figure 7: A heatmap displaying the z-scores of microbial pathways from the second sample set. Samples are clustered by sample type and subject type. Within sample type, yellow represents mucosal aspirates, brown represents fecal samples, and purple represents lavage aspirates. Within subject type, green represents polyp free samples, blue represents serrated polyp samples, and red represents tubular adenoma samples. A total 507 pathways were identified.

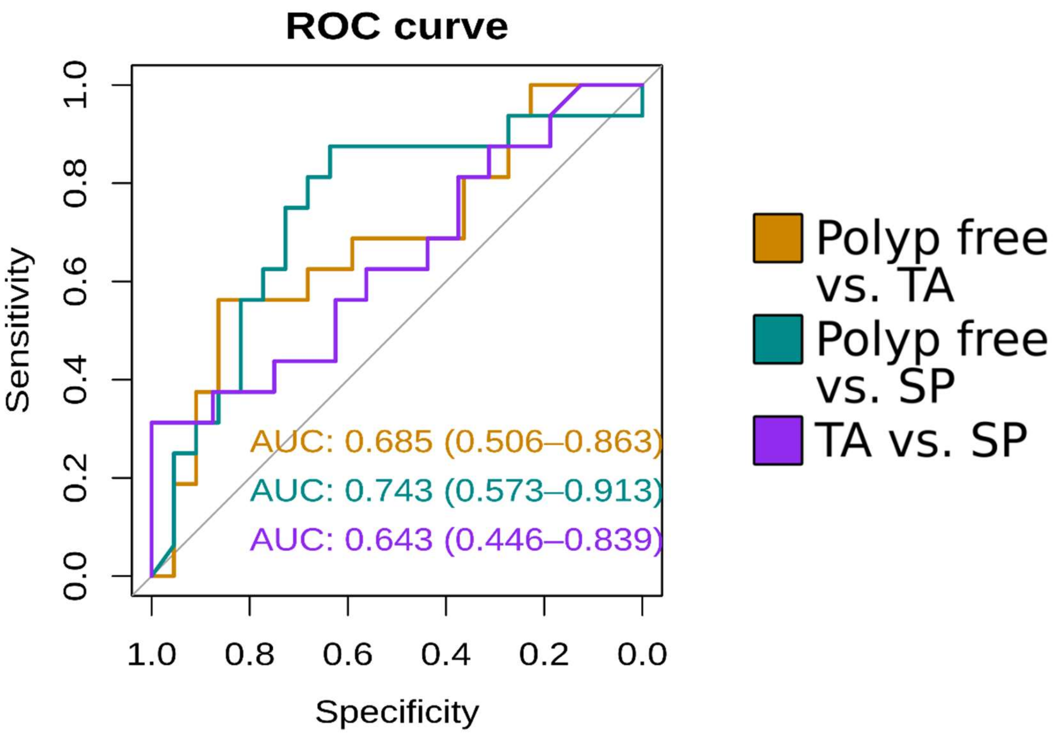

Supplementary Figure 8: A receiver operating characteristic (ROC) curve illustrating the true positive rate (Sensitivity, y-axis) versus the false positive rate (Specificity, x-axis) produced by Random Forest classification of functional pathways in second sample set mucosal aspirates. The area under the curve (AUC) value for each Random Forest is displayed with the 90% confidence interval.

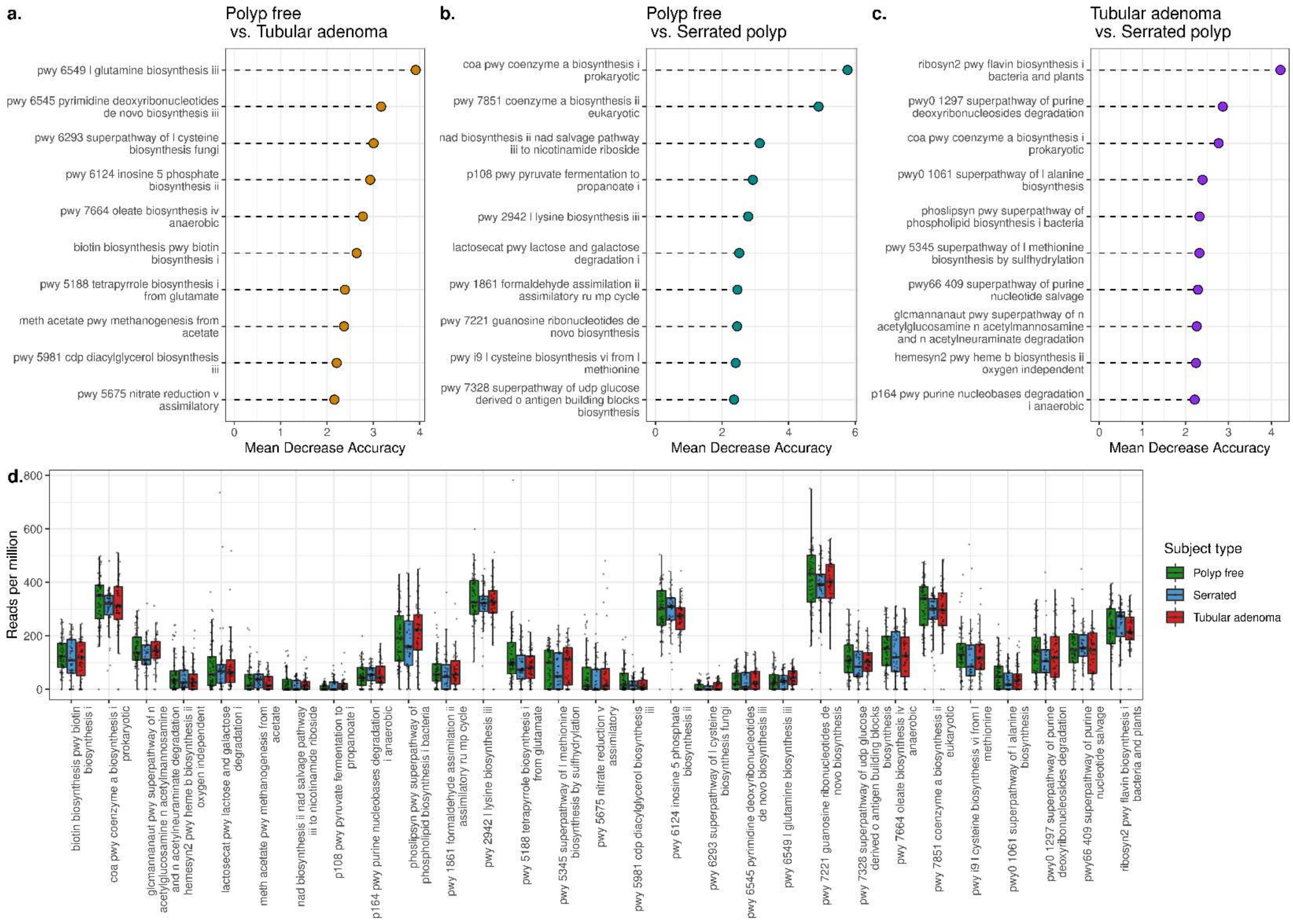

Supplementary Figure 9: **A-C)** The top ten variables of importance for each pairwise random forest classification of functional pathways in second sample set mucosal aspirates. Variables are sorted by their mean decrease in accuracy, with larger means contributing greater to Random Forest performance. **D)** Box plots displaying the functional pathway abundances (in reads per million) of the top variables of importance as determined by Random Forest. Each point is one sample, with multiple samples per individual. The center line within each box defines the median, boxes define the upper and lower quartiles, and whiskers define 1.5x the interquartile range.

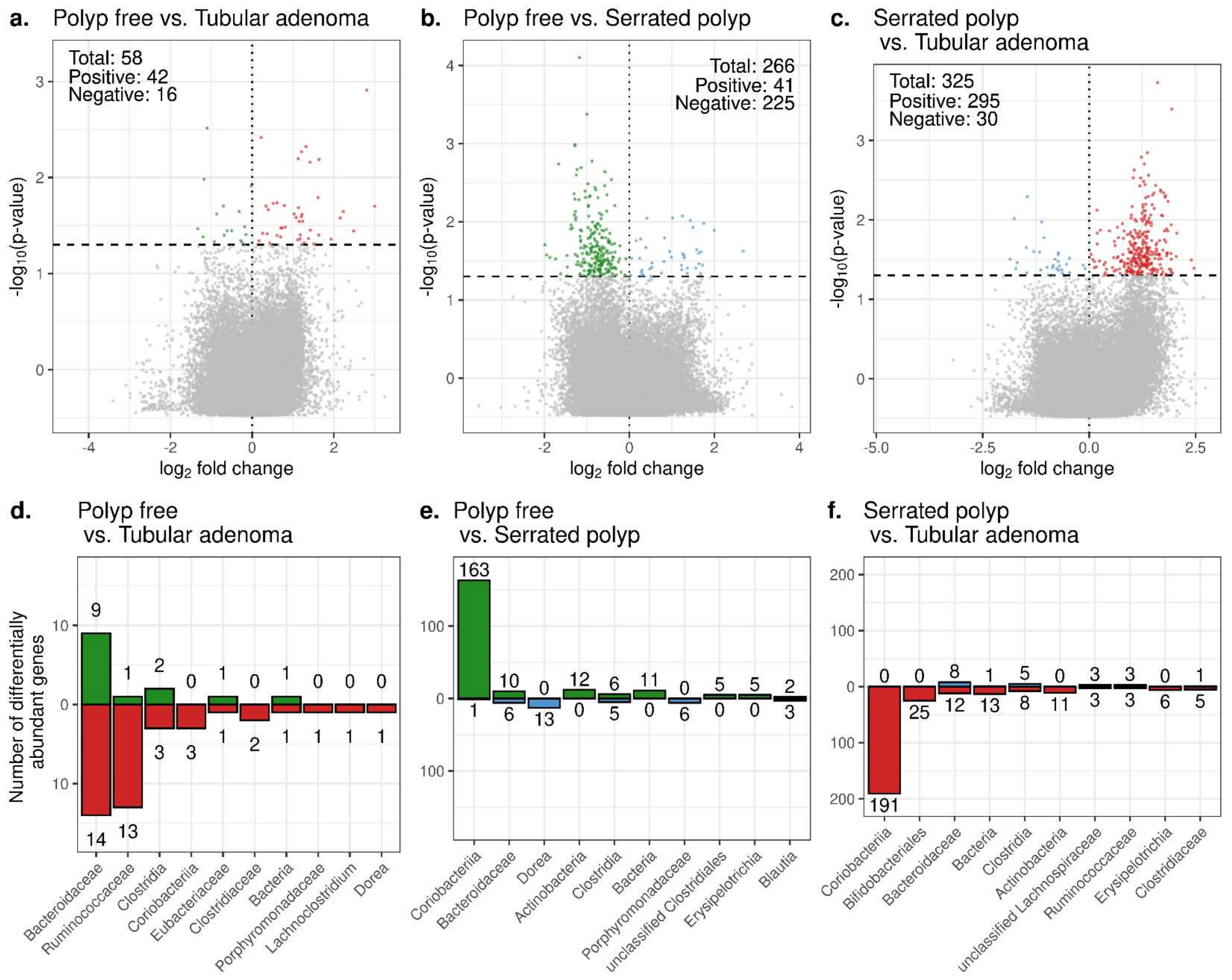

Supplementary Figure 10: **A-C**) Volcano plots illustrating the differentially abundant microbial genes within mucosal aspirate samples from the second sample set before FDR correction (Kruskal-Wallis: $p < 0.05$). The horizontal and vertical lines denote a significance threshold of $p = 0.05$, and zero $\log_2$fold change, respectively. Points are colored to denote the subject type in which the gene was more abundant, with green referring to genes more abundant in polyp-free samples, red for tubular adenomas, and blue for serrated polyps. The number of total, negative fold-change, and positive-fold change genes with an unadjusted p-value < 0.05 are displayed within each graph. **D-F**) The number of differentially abundant genes per taxon for each subject type comparison. Only the top ten taxa with the most differentially abundant genes are shown.

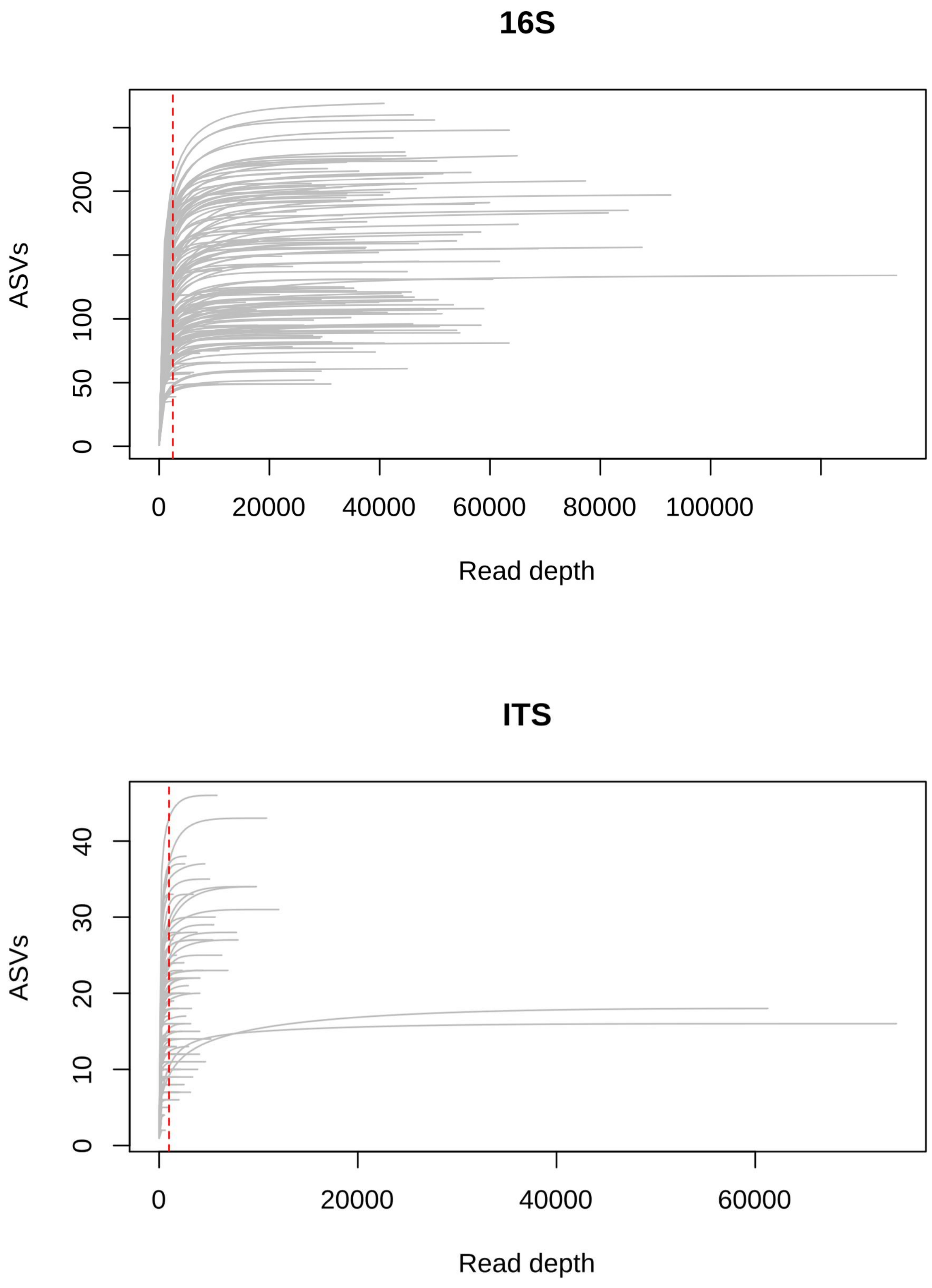

Supplementary Figure 11: Rarefaction curves of 16S (top) and ITS (bottom) amplicons from the first sample set. The x-axis is the read depth of each sample, with each line representing one sample. The y-axis is the number of unique ASVs per sample. The dotted red line represents the minimum required read depth for analysis inclusion.

For 16S sequencing, this was determined at 2,500 high-quality, taxonomically annotated sequences, and for ITS it was 1,000 high-quality, taxonomically annotated sequences.
