# Supplementary material for: Distinct colon mucosa microbiomes associated with tubular adenomas and serrated polyps
Source: NPJ Biofilms Microbiomes. 2022 Aug 29;8:69. doi: 10.1038/s41522-022-00328-6 (PMC9424272; doi:10.1038/s41522-022-00328-6)
Supplement: Supplementary file 4 — Reporting Summary [file 41522_2022_328_MOESM4_ESM.pdf]

nature portfolio

Corresponding author(s): Julio Avelar-Barragan
Katrine Whiteson

Last updated by author(s): Mar 2, 2022

Reporting Summary

Nature Portfolio wishes to improve the reproducibility of the work that we publish. This form provides structure for consistency and transparency in reporting. For further information on Nature Portfolio policies, see our Editorial Policies and the Editorial Policy Checklist.

Statistics

For all statistical analyses, confirm that the following items are present in the figure legend, table legend, main text, or Methods section.

n/a	Confirmed	
☐	☒	The exact sample size (n) for each experimental group/condition, given as a discrete number and unit of measurement
☐	☒	A statement on whether measurements were taken from distinct samples or whether the same sample was measured repeatedly
☐	☒	The statistical test(s) used AND whether they are one- or two-sided<br>*Only common tests should be described solely by name; describe more complex techniques in the Methods section.*
☐	☒	A description of all covariates tested
☐	☒	A description of any assumptions or corrections, such as tests of normality and adjustment for multiple comparisons
☐	☒	A full description of the statistical parameters including central tendency (e.g. means) or other basic estimates (e.g. regression coefficient) AND variation (e.g. standard deviation) or associated estimates of uncertainty (e.g. confidence intervals)
☐	☒	For null hypothesis testing, the test statistic (e.g. F, t, r) with confidence intervals, effect sizes, degrees of freedom and P value noted<br>*Give P values as exact values whenever suitable.*
☒	☐	For Bayesian analysis, information on the choice of priors and Markov chain Monte Carlo settings
☐	☒	For hierarchical and complex designs, identification of the appropriate level for tests and full reporting of outcomes
☒	☐	Estimates of effect sizes (e.g. Cohen's d, Pearson's r), indicating how they were calculated

Our web collection on statistics for biologists contains articles on many of the points above.

Software and code

Policy information about availability of computer code

Data collection

Qiime2 v2019.1 - Used to acquire amplicon sequence variant tables for 16S and ITS sequencing.
Greengenes database v13.8 - Used to assign taxonomy to 16S data within Qiime2.
UNITE database v8.0 - Used to assign taxonomy to ITS data within Qiime2.
BBMap v39.79 - Used to remove Illumina adaptors and demultiplex raw shotgun data.
PRINSEQ++ v1.2 - Used to quality filter shotgun sequences.
BowTie2 v2.3.5.1 - Used for the alignment of shotgun sequences.
IGGSearch v1.0 - Used to assign taxonomy to shotgun reads.
HUMAnN v3.0.1- Used to acquire pathway abundances of shotgun data.
ChocoPhlAn v296 - Database used for HUMAnN.
UniRef90 v201901b - Database used for HUMAnN.
MEGAHIT v1.1.1 - Used to assemble shotgun reads into contiguous sequences.
Prodigal v2.6.3 - Used to identify open reading frames within contiguous sequences.
eggNOG mapper v2.0 - Used to annotate open reading frames.
eggNOG v5.0 - Database used by eggNOG mapper.
MicrobeCensus v1.1.1 - Used to normalize gene count abundances of annotated shotgun data.

Data analysis

R v3.6.3 - Used for the analysis of 16S, ITS, and shotgun data.
EcolUtils v0.1 - Used to permutationally rarefy amplicon data.
Vegan v2.5-6 - Used to perform alpha, beta, and PERMANOVA analyses.
nlme v3.1-148 - Used to perform linear mixed effects model hypothesis testing.
ANCOM v2.1 - Used for differential abundance testing while accounting for random effects.
IHW v1.14.0 - Used for adjusting the FDR values obtained by Kruskal-Wallis testing.
rfPermute v1.9.3 - Used to perform Random Forest.

pROC v1.18.0 - Used to obtain ROC curves and AUC values from Random Forests.

For manuscripts utilizing custom algorithms or software that are central to the research but not yet described in published literature, software must be made available to editors and reviewers. We strongly encourage code deposition in a community repository (e.g. GitHub). See the Nature Portfolio guidelines for submitting code & software for further information.

Data

Policy information about availability of data

All manuscripts must include a data availability statement. This statement should provide the following information, where applicable:

- Accession codes, unique identifiers, or web links for publicly available datasets
- A description of any restrictions on data availability
- For clinical datasets or third party data, please ensure that the statement adheres to our policy

All code for data processing and analysis is available on GitHub at: https://github.com/Javelarb/ACS_polyp_study. Additional data and materials are available upon reasonable request.

Field-specific reporting

Please select the one below that is the best fit for your research. If you are not sure, read the appropriate sections before making your selection.

☒ Life sciences ☐ Behavioural & social sciences ☐ Ecological, evolutionary & environmental sciences

For a reference copy of the document with all sections, see nature.com/documents/nr-reporting-summary-flat.pdf

Life sciences study design

All studies must disclose on these points even when the disclosure is negative.

Sample size	We originally sought to recruit enough subjects to ensure we had a minimum of 15 subjects with serrated polyps, 15 with tubular adenomas, and 15 without polyps. This limit was based on the need for an adequate sampling of serrated polyps and their frequency in the surveillance colonoscopy patient population. We determined that a minimum of 15 individuals per group would provide sufficient PERMANOVA power to detect significant effect sizes of at least 0.3% in gut microbiome composition. This calculation was obtained using the 'micropower' package in R, which simulates microbiome data. Our present study exceeds our original sample collection goals by more than double.
Data exclusions	No data were excluded from our study.
Replication	We did not attempt to reproduce this study. Doing so would require collecting additional human samples, which would require additional IRB approval.
Randomization	Randomization was not performed during sample collection, as there was no treatment group. Our study aims to characterize the gut microbiome of individuals with and without colorectal polyps.
Blinding	Blinding of study participants was not necessary for our study, since we did not perform an active intervention or treatment.

Reporting for specific materials, systems and methods

We require information from authors about some types of materials, experimental systems and methods used in many studies. Here, indicate whether each material, system or method listed is relevant to your study. If you are not sure if a list item applies to your research, read the appropriate section before selecting a response.

Materials & experimental systems

n/a	Involved in the study	
☒	☐	Antibodies
☒	☐	Eukaryotic cell lines
☒	☐	Palaeontology and archaeology
☒	☐	Animals and other organisms
☐	☒	Human research participants
☒	☐	Clinical data
☒	☐	Dual use research of concern

Methods

n/a	Involved in the study	
☒	☐	ChIP-seq
☒	☐	Flow cytometry
☒	☐	MRI-based neuroimaging

Human research participants

Policy information about studies involving human research participants

Population characteristics	Our population study was male dominated, had a median age of 60+, and a median BMI of 25 kg/m^2. Our largest ethnic category was 'White'. A table containing the population has been included in the main text (Table 1).
Recruitment	Recruitment procedure:<br>Individuals who presented for colonoscopy with indications of screening for, or a prior history of, colorectal polyps were asked to participate in the study. Written and informed consent was obtained from each subject and was required for participation. Subjects who were pregnant, had taken antibiotics within 6 weeks of colonoscopy, or with known inflammatory bowel diseases, were excluded.<br><br>Biases:<br>The recommended screening age for colorectal cancer is 45, thus most of our samples are from subjects older than 45. Age has been known to impact the composition of the gut microbiome. According to PERMANOVA analyses, we observed that age significantly explained 1 to 5% of the variance in microbiome composition.
Ethics oversight	This study was approved by the Institutional Review Board (IRB) of the University of California, Irvine (HS# 2017-3869).

Note that full information on the approval of the study protocol must also be provided in the manuscript.
